# Weaning failure from mechanical ventilation: a scoping review of the utility of ultrasonography in the weaning process

**DOI:** 10.1016/j.bja.2025.02.024

**Published:** 2025-03-27

**Authors:** Patrick Sepúlveda, Adrián Gallardo, Ricardo Arriagada, Bruno Souza, Nicolò Patroniti, Denise Battaglini

**Affiliations:** 1 Servicio de Medicina Física Y Rehabilitación, Hospital de La Serena, La Serene, Chile; 2 Grupo de Investigación MoVICU, Chile; 3 Servicio de Kinesiología, Clínica Modelo de Morón, Buenos Aires, Argentina; 4 Departamento de Ciencias de la Salud, Universidad Nacional de la Matanza, Kinesiología y Fisiatría, San Justo, Argentina; 5 Unidad de Paciente Crítico Adulto, Hospital Las Higueras de Talcahuano, Talcahuano, Chile; 6 Escuela de Kinesiología Universidad San Sebastián, Sede Tres Pascualas, Concepción, Chile; 7 Hospital Universitário Antônio Pedro. Universidade Federal Fluminense, Brazil; 8 Department of Surgical Sciences and Integrated Diagnostics (DISC), University of Genoa, Genoa, Italy; 9 Anesthesia and Intensive Care, IRCCS Ospedale Policlinico San Martino, Genoa, Italy

**Keywords:** critical care, extubation, intensive care, mechanical ventilation, post-extubation failure, ultrasonography, weaning

## Abstract

**Background:**

Weaning failure has been associated with increased hospital stay and higher mortality. Identification of the risk factors that may affect weaning outcome is paramount. Ultrasonography is an excellent tool for pulmonary and diaphragmatic monitoring during mechanical ventilation, allowing real-time evaluation of anatomical structures and function. We performed a scoping review to highlight the usefulness and limitations of ultrasonography as a tool for detecting weaning failure.

**Methods:**

The Joanna Briggs Institute recommendations, the PRISMA Extension for Scoping Reviews (PRISMA-ScR) checklist, and the methodological framework by Arksey and O'Malley were followed. We searched PubMed, Scopus, and Cochrane databases for observational and randomised studies published from inception to August 12, 2024. Inclusion criteria were articles written in English, intensive care unit setting, mechanical ventilation, adults, and those that described a measure for burden of weaning failure using ultrasonography.

**Results:**

The search revealed 3573 records. After removal of duplicates, 3117 articles were screened for potential inclusion, of which 89 articles were finally included. These comprised six clinical trials, 80 observational prospective studies, and three retrospective studies. In total, 6841 subjects were included, with a weaning failure rate of 28.2%. The parameters most associated with weaning failure were higher ratio of early diastolic mitral inflow velocity to early diastolic septal mitral annulus velocity, diaphragmatic excursion, diaphragm thickening fraction, diaphragmatic rapid shallow breathing index, ratio between ventilatory frequency, diaphragmatic displacement, excursions, and contraction velocity on coughing. Loss of aeration, pulmonary oedema, and pleural effusion detected with ultrasound were associated with weaning failure and airway obstruction caused by post-extubation laryngeal oedema.

**Conclusions:**

Ultrasound represents a valuable tool for optimising the weaning process. It enables precise assessment of lung function and diaphragmatic performance, underscoring the need for its implementation in ICU setting.

**Editor's key points**

• Weaning from mechanical ventilation is crucial but challenging, with many patients experiencing difficulties; however, the specific role of ultrasonography in predicting weaning failure remains unclear.
• This scoping review highlights the utility of ultrasonography in identifying weaning failure owing to cardiovascular, diaphragmatic, respiratory, and airway issues, emphasising its potential to enhance clinical decision-making.
• The findings suggest that integrating ultrasound into the weaning process could improve outcomes. Future research should focus on standardising its use and exploring its long-term impacts on patient recovery.

Weaning from mechanical ventilation (MV) is a crucial part of care for patients on invasive ventilatory support, consuming around 40–50% of the total time spent on ventilation. However, 20% of patients undergoing MV show difficulties in this process and are characterised as patients with difficult or prolonged weaning, with around 15.6% reporting weaning failure.^1^^,^^2^

To address these challenges, Tobin^3^ proposed a series of steps in the process of weaning from invasive MV. These steps include treating acute respiratory failure (ARF), assessing the possibility of weaning, evaluating readiness to wean, conducting spontaneous breathing trial (SBT), extubating, and re-intubating. This structured approach was validated by a panel of expert developing the first guidelines about weaning from MV in 2007.^4^ The understanding of weaning has progressed significantly over the years. In 2017, the WIND study offered a new perspective by defining weaning based on the need for reintubation within 48 h or 7 days after extubation.^5^^,^^6^ More recently, the WEAN−SAFE study,^2^ which involved 50 countries, found that 77.1% of patients underwent at least one separation attempt, and 65% were successfully weaned from ventilation at day 90. The first separation attempt was initiated at day 1, but 22.4% had a delay of about 5 days in initiating first separation attempt. The findings revealed that 64.7% of patients experienced short weaning (≤1 day), 10.1% had intermediate weaning (2–6 days), 9.6% faced prolonged weaning (≥7 days), and 15.6% experienced weaning failure.^2^

Weaning failure has various underlying mechanisms, with several risk factors coming into play, such as age, prior home MV, reason for the ventilation, and underlying disorders.^7^

In recent years, the use of ultrasound has gained attraction as a valuable tool in assessing patients during the weaning process. It has proven effective in detecting specific features associated with weaning failure, including diaphragmatic dysfunction, presence of extensive pleural effusions, laryngeal oedema, acute pulmonary oedema, and inadequate pulmonary aeration.^8^ Advancements in ultrasonography have allowed for its integration into weaning protocols, providing real-time insights that enhance clinical decision-making. For example, ultrasound can evaluate diaphragmatic movement and lung aeration, identifying subtle changes that might be overlooked through traditional assessments. Furthermore, it can help detect fluid overload or other pulmonary complications that could impede successful weaning.^9^, ^10^, ^11^ Despite these advancements, the exact role of ultrasonography in predicting weaning failure is still not fully understood, highlighting the need for further research and standardisation.^8^

This scoping review aims to explore the utility of ultrasonography in predicting weaning failure in patients receiving invasive MV.

## Methods

### Study design

This is a scoping review that follows the Joanna Briggs Institute recommendations,^12^ the PRISMA Extension for Scoping Reviews (PRISMA-ScR) checklist,^13^ and the methodological framework by Arksey and O'Malley.^14^ As this review involves analysis of previously published studies, no ethical approval was required. However, care was taken to adhere to ethical standards in reporting and data interpretation.

The primary aim of this review was to investigate how ultrasonography can assist in predicting weaning failure from MV. Specifically, we examined parameters related to: (1) echocardiography (weaning failure from cardiovascular origins); (2) diaphragm ultrasound (weaning failure from diaphragmatic dysfunction); (3) lung ultrasound (weaning failure from respiratory causes); and (4) ultrasound of upper airways (weaning failure from airway obstruction). Moreover, we aimed at exploring how these ultrasonographic parameters were associated with successful weaning and to identify areas where further research is needed, including the integration of ultrasound findings into clinical workflow and the implications for future studies.

### Eligibility criteria

Peer-reviewed journal papers were included if they were published from inception to August 12, 2024, written in English, involved human participants, developed in ICU setting, included adult patients who were mechanically ventilated, and described a measure for burden of weaning failure using ultrasonography. Randomised controlled trials (RCTs), observational studies, and retrospective studies were included. Exclusion criteria were studies not focused on weaning failure or those that used non-ultrasonographic methods for assessment, patients aged <18 yr, and case report studies.

## Search and screening strategy

Two authors (DB and PS) identified the strings with the MeSH terms and performed the search strategy in three databases: PubMed, Cochrane, and Scopus. The search was reviewed by the other authors according to the Peer Review of Electronic Search Strategies (PRESS) checklist.^13^ The following MeSH terms and Boolean operators were adopted: ‘([weaning OR liberation] AND [mechanical ventilation] AND [ultrasonog∗ OR ultrasound])’ and adapted to the searched database. No filters were applied to the search.

After removing duplicates, the titles and abstracts of the selected articles were screened by two authors (DB and PS) for potential inclusion. All authors assessed the complete texts, abstracts, and titles of every selected article. Disagreements were solved by consensus. Systematic reviews and meta-analyses were screened looking for articles potentially missed during our preliminary search.

### Data extraction and presentation

Two reviewers (PS and DB) created a data-charting form with variables to extract. In an iterative process, the two reviewers separately charted the data, discussed the findings, and updated the data-charting form on a regular basis.

Quantitative, qualitative, and mixed-method studies were included to consider different aspects of measuring weaning burden. Papers were excluded if they did not fit into the conceptual framework of the study, according to the inclusion criteria.

Studies were categorised on the types of behaviour they examined, settings, population, and study design, and also the measures used and broad findings. The data extracted comprised year of publication, first author, design of the study, country of origin, number of included patients, number of patients who failed weaning, reason for weaning failure, type ultrasonographic assessment (e.g. heart, lung, diaphragm), parameter tested using ultrasound for assessment of weaning failure, and quantitative ultrasound data. Considering the ‘scoping’ nature of this review, data were reported mainly as descriptive synthesis.

### Definitions of ultrasonographic variables

Table 1 summarises the definitions adopted for ultrasonographic parameters associated with weaning failure.

**Table 1:** Definitions adopted for ultrasonographic parameters associated with weaning failure.

*Parameter*	Definition	Normal value
***Echocardiography***		
*Mitral septal E/E′ ratio*	Defined as the ratio of early diastolic mitral inflow velocity (E) to the early diastolic velocity of the mitral annulus as measured by tissue Doppler imaging (E′). It serves as an estimate of left ventricular filling pressures.	A higher E/E′ ratio indicates elevated left atrial pressure and suggests diastolic dysfunction or heart failure. Normal value <8. Values 8–15 indicate mild to moderate diastolic dysfunction, and values >15 indicate severe dysfunction.
*E/A ratio*	The ratio of the early diastolic mitral inflow (E) to the late diastolic mitral inflow (A). A decreased E/A ratio indicates impaired relaxation. Diastolic dysfunction is significant as it can lead to heart failure with preserved ejection fraction, which affects fluid dynamics	Normal value is >1 during diastole. Normal deceleration time is <220 ms. Higher values indicate impaired relaxation.
*Left atrial area (LAA)*	Defined as the measurement of the size of the left atrium. It serves as a marker for left atrial pressure and overall cardiac function. It can be measured using two-dimensional echocardiography, typically in the apical four-chamber view, by tracing the left atrial border.	Normal value is <20 cm^2^. Greater values may indicate left atrial enlargement, often associated with diastolic dysfunction.
*Fixed rightward curvature of the interatrial septum*	Defined as structural abnormality of the septum between the right and left atria, often associated with elevated right atrial pressure.	No standard exists as it may vary according to anatomical differences.
***Diaphragm ultrasound***		
*Diaphragmatic excursion (DE)*	Defined as the distance the diaphragm moves during breathing cycles (measured in cm).	The normal range of DE is >2 cm.
*Diaphragm thickening fraction (DTF)*	Defined as a surrogate of diaphragmatic function and muscle strength, assessed as the change in diaphragm thickness during inspiration compared with its thickness at rest. It is calculated using the following formula: dtf = (thickening at inspiration − thickness at rest)/thickness at rest/100.	A normal value is generally >20% during spontaneous breathing.
*Diaphragmatic-rapid shallow breathing index (D-RSBI)*	Defined as the ratio between the respiratory rate and the ultrasonographic displacement, assessing the efficiency of the diaphragm in relation to the breathing rate.	Normal value is generally considered as <100 bpm mm^−1^.
*Diaphragmatic displacement (DD)*	Defined as the amount of vertical movement of the diaphragm during inspiration, assessed through M-mode ultrasound. DD is estimated with the formula = diaphragm position at end-expiration – diaphragm position at end-inspiration.	Normal values for DD during unassisted breathing are typically >1 cm.
***Lung ultrasound***		
*Lung ultrasound score (LUS)*	By assigning scores to different lung regions based on ultrasound findings, it assesses the degree and existence of lung aeration. There are 12 areas in the lung (six anterior and six posterior). Each region is scored from 0 to 3 based on the following criteria: 0: normal aeration (A-lines present, no B-lines); 1: moderate loss of aeration (one to two B-lines); 2: severe loss of aeration (three to five B-lines); 3: complete loss of aeration (consolidation).	Total score: the LUS ranges from 0 to 36, with higher scores indicating greater lung pathology.
***Ultrasound of upper airways***		
*Air column width measurement during cuff deflation*	Linear ultrasound transducer is placed over the upper airway while the tube cuff is inflated and then gradually deflated.	The normal pharyngeal diameter is 10–15 mm. The normal laryngeal diameter is around 10–12 mm. The normal air column diameter in the trachea is 6–10 mm.

## Results

**Fig 1:**
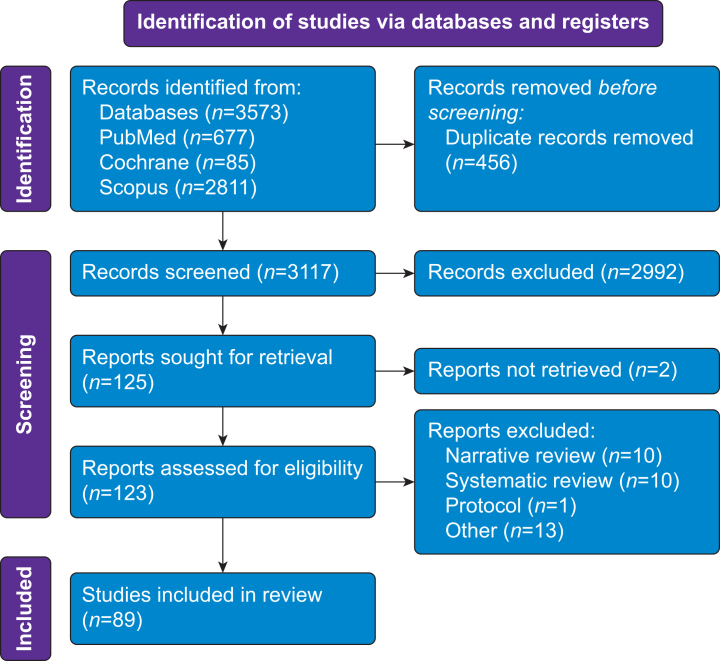
PRISMA flow-chart of inclusion. From Page MJ, McKenzie JE, Bossuyt PM *et al.* The PRISMA 2020 statement: an updated guideline for reporting systematic reviews. *BMJ* 2021; **372**: n71.

The search revealed 3573 records (677 PubMed, 2811 Scopus, 85 Cochrane). After removal of duplicates (456), 3117 articles were screened for potential inclusion, and 89 articles were finally included. The complete articles selection process is shown in Figure 1.

The included studies comprised six clinical trials, 80 observational prospective studies, and three retrospective studies. The total number of subjects included was 6841. The mean age of the included subjects ranged from 22 to 89 yr. The included studies were mainly from China, India, and France.

A total of 1900 (28.2%) failed weaning (95 subjects were found to be missing weaning outcome data). Weaning failure from cardiac origin was identified by ultrasound in nine studies, diaphragmatic dysfunction in 63 studies, loss of aeration in 16 studies, pleural effusion in one study, laryngeal stridor post-extubation in one study. Table 2 summarises the main ultrasonographic parameters and cutoffs associated with weaning failure, according to evidence^15^, ^16^, ^17^, ^18^. Table 3 presents the randomised clinical trials included for review^19^, ^20^, ^21^, ^22^, ^23^, ^24^, and Supplementary material S1 presents the observational studies identified from the search.

**Table 2:** Main ultrasonographic parameters and cutoffs associated with weaning failure. See Table 1 for definitions. AUROC, area under receiver operating curve; CI, confidence interval; DD, diaphragmatic displacement; DE, diaphragm excursion; DTF, diaphragmatic thickening fraction; IQR, interquartile range; IVC, inferior vena cava; LUS, lung ultrasound; OR, odds ratio; VF, ventilatory frequency; RSBI, rapid shallow breathing index; SBT, spontaneous breathing trial; WIPO, weaning-induced pulmonary oedema.

*Causes of weaning failure*	Associated parameters	Cutoff values
***Cardiovascular origin***	- Higher ratio of early diastolic mitral inflow velocity to early diastolic septal mitral annulus velocity (mitral septal E/E′) - Higher diastolic dysfunction - Left ventricular diastolic dysfunction - Raised left atrial pressure indicated by interatrial septal fixed rightward curvature - Tissue Doppler imaging assessing the mitral annulus - Left ventricular relaxation (e’ wave) - Left ventricular filling pressure (E/e’ ratio)	- e/E′ values: 10.9 (IQR 8.7–18.0) - Left atrial area: 25.0 (IQR 19.3–32.0) - Fixed rightward curvature of the interatrial septum: median of 7 (OR 12.95, 95% CI 2.73–61.41) - Peak A wave <100 cm s^−1^ - E/Ea >14 - IVC max >17 mm - Peak E-wave velocity: 95 cm s^−1^ (75–115) *vs* 85 cm s^−1^ (61–100), *P*<0.001 - E-wave deceleration time: 128 ms (89–163) *vs* 147 ms (109–206), *P*<0.001 - E velocity ≥0.6 m s^−1^ at baseline and E ≥1.2 m s^−1^ during SBT: 100% specificity, 15% sensitivity; 91% sensitivity to identify failed SBT owing to WIPO
***Diaphragmatic dysfunction***	- Diaphragmatic excursion (DE) - Diaphragm thickening fraction (DTF) - Diaphragmatic-RSBI (D-RSBI) - Ratio between ventilatory frequency (VF) and diaphragmatic displacement (DD)	- DE: ≤1.1 cm - DTF: <20% - DE-RSBI >1.38 bpm mm^−1^ - DTF-RSBI >78.1 bpm %^−1^ - VF/DD ratio: sensitivity 87.5%, specificity 92.31 - VF/DTF ≤0.81: sensitivity 87.7%, specificity 75% (AUROC 0.762)
***Respiratory cause (loss of aeration, pulmonary oedema, pleural effusion)***	- Lung ultrasound score (LUS) - Alveolo-interstitial syndrome (increased B-line artifacts) - Disappearance of peripheral parenchymal lesion (C-lines)	- Anteroposterior LUS score: >7 - Conventional LUS score: >10 - Another reported cutoff: >17 - Maximal interpleural distance for moderate to large effusions: ≥15 mm; for large effusions ≥25 mm
***Airway obstruction***	- Laryngeal stridor post-extubation - Air column width measurement during cuff deflation	- Air column diameter: <4.5 mm (7–8% stridor rate) - Diameter >6.4 mm (no stridor)

**Table 3:** Randomised controlled trials on weaning and ultrasonography.

First author	Year	Country	Number of patients	Randomisation	Weaning failure (*No*. of patients)	Principal measures and results
**Allam MG**^19^	2023	Saudi Arabia	200	Patients were randomised into two groups (control group, weaned by conventional parameters, 100; weaned by echographic weaning parameters, 100)	50	Measured in the group weaned with echography showed that diaphragmatic excursion (DE) is weaned at a cutoff of 1 cm, diaphragm thickening fraction (DTF): both sides ranged from 0.7 to 1.5 in males and 0.6 to 1.6 in females. This group of patients was weaned upon resolution of three ultrasonic findings: (1) removal of B-line artifacts; (2) disappearance of C-lines; and (3) the dynamic air-bronchogram has disappeared. Control group had a considerably greater rate of weaning failure, defined as reintubation before 48 h.
**Zeid D**^20^	2021	Egypt	80	Patients were divided in two groups who met the readiness criteria to start spontaneous breathing trial (SBT) either on: pressure support ventilation (PSV) for 30 min or T-piece for 120 min.	20	Early diastolic mitral inflow velocity divided by early diastolic septal mitral annulus velocity (mitral septal E/E′). In the PSV group, the threshold value for mitral septal E/E′ was ≥6.1, with a sensitivity of 81%, specificity of 84.2%, and area under curve (AUC) of 0.73 in predicting weaning failure. The cutoff value for inferior vena cava (IVC) distensibility index is ≥66.5%, with 100% sensitivity, 68.4% specificity, and an AUC of 0.85. In the T-piece group, a mitral septal E/E′ cutoff value of ≥5.8 was found to have a sensitivity of 83%, specificity of 90.9%, AUC of 0.83, and IVC collapsibility index. The cutoff value is ≥45.5%, with 72% sensitivity, 86% specificity, and an AUC of 0.73. Mitral septal E/E′ may indicate weaning-induced diastolic dysfunction. IVC is a crucial factor in predicting weaning failure.
**McCool DF**^22^	2020	USA	32	Patients were randomised into two groups (control, 15; diaphragm ultrasound, 17).	3	The intervention group had a substantially shorter duration from ultrasonography to extubation than the control group in patients with a Δtdi% ≥30% (4.8 standard deviation, SD=8.4 *vs* 35.0 SD=41.0 h, *P*=0.04). The period from ultrasonography to extubation was shorter in participants with a typically functioning diaphragm (Δtdi% ≥30%) than in those with diaphragm dysfunction (Δtdi% <30%) (23.2 SD=35.2 *vs* 57.3 SD=52.0 h, *P*=0.046). Combining the intervention and control groups, a value of Δtdi% ≥30% for extubation success at 24 h resulted in sensitivity, specificity, positive predictive value (PPV), and negative predictive value (NPV) of 90.9%, 86.7%, 90.9%, and 86.7%, respectively.
**Mowafy S**^21^	2018	Egypt	106	Patients were divided in two groups: Group 1, traditional rapid shallow breathing index (RSBI) (RSBI <105 predicts successful weaning); Group II, weaning based on the result of diaphragmatic RSBI (D-RSBI) (D-RSBI <1.3 predicts successful weaning)	33	D-RSBI at 30 min had the highest diagnostic accuracy for predicting weaning success, with a threshold of <1.6 bpm mm^−1^ at 30 min. The results showed 97.3% sensitivity, 93.9% specificity, 97.1% PPV, 93.9% NPV, and 96.2% accuracy. D-RSBI, which guides release from mechanical breathing, has higher diagnostic accuracy than regular RSBI, especially when checked 30 min after starting SBT.
**Osman A**^23^	2017	Egypt	68	Patients were divided between groups of: success and failure of weaning	18	The DE, DTF, and lung ultrasound score (LUS) were all assessed. DE: patients with diaphragmatic E more than 22 mm weaned successfully, but all patients with diaphragmatic E less than 11 mm failed to wean. DTF: all patients with DTF values greater than 31% weaned successfully, but all patients with DTF values less than 29% failed to wean. LUS: all patients with a LUS more than 17 (line A) failed weaning, whereas those with a score less than 13 (line B) were successful. Diaphragmatic and lung ultrasonography give quick and noninvasive indicators for the weaning process compared with other standard measures such as blood gases and respiratory mechanics.
**Soummer A**^24^	2012	France	100	Patients were divided between groups of success and failure of weaning	29	Lung ultrasonography and echocardiography were performed before and after a 60-min SBT, and 4 h after extubation. Only the group that failed weaning experienced decreased lung aeration during the successful SBT, with lung ultrasonography scores increasing from 15 (13;17) to 19 (16;21), *P*<0.01. End-SBT lung ultrasonography scores were considerably higher in the weaning failure group than in the success group: 19 (16;21) *vs* 10 (7;13), respectively (*P*<0.001) and predicted post-extubation discomfort with an area under the receiver operating characteristic curve of 0.86. Lung ultrasonography analysis of aeration changes during a successful spontaneous breathing attempt can reliably predict post-extubation suffering.

## Discussion

**Fig 2:**
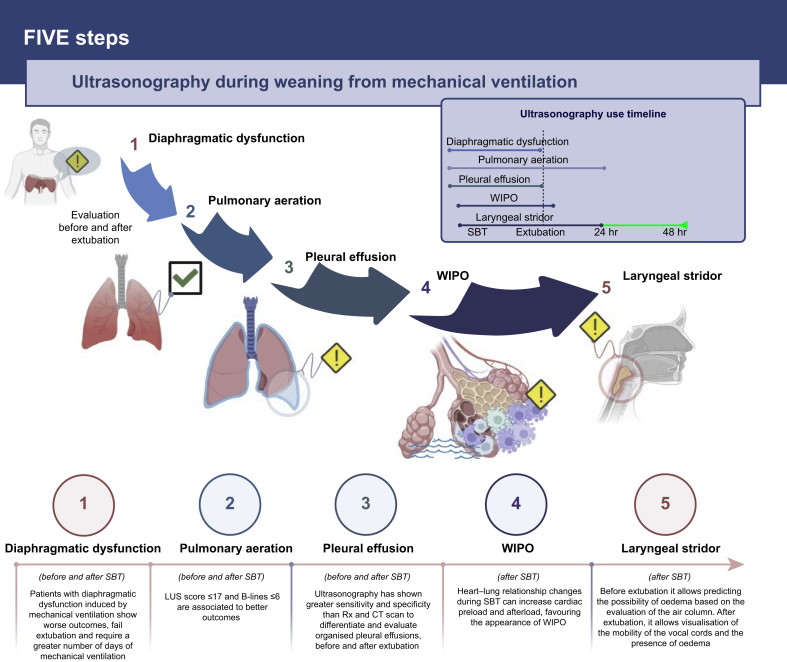
Five key steps of ultrasonography in weaning from mechanical ventilation. This figure shows the main domains identified by lung ultrasound to facilitate clinical decision-making in weaning process. LUS, lung ultrasound score; SBT, spontaneous breathing test; WIPO, weaning-induced pulmonary oedema; X-Ray, radiography.

**Fig 3:**
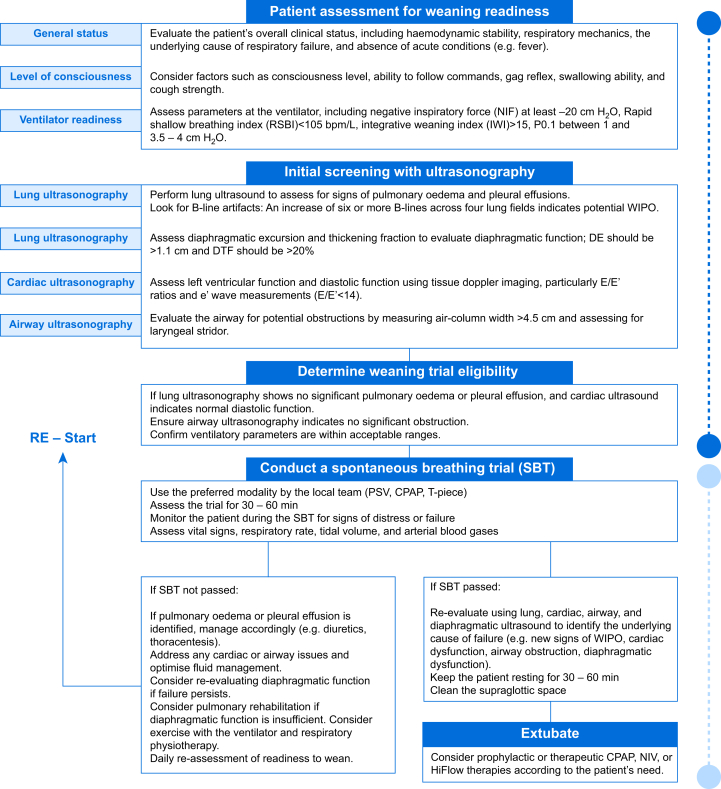
Proposed algorithm to integrate ultrasonography in the weaning process. This figure presents a possible algorithm for integrating ultrasound into the weaning process. CPAP, continuous positive airway pressure; IWI, integrative weaning index; NIF, negative inspiratory force; NIV, noninvasive ventilation; PSV, pressure support ventilation; RSBI, rapid shallow breathing index.

Our findings reveal that ultrasound is a useful tool for identifying weaning failure from cardiovascular, diaphragmatic, respiratory, and airway origin. The strengths of our scoping review lie in its comprehensive approach to synthesising diverse ultrasound parameters, providing insights into the multifactorial nature of weaning failure, and highlighting the potential for ultrasound to enhance clinical decision-making by identifying physiological challenges that may impede successful extubation. Although there are still several areas for further study and real-world implementation, the incorporation of ultrasonography into the process of weaning from MV is encouraging. We suggest the following approaches to improve the use of ultrasonography in clinical practice, as reported in Fig 2, Fig 3.

### Weaning failure from cardiovascular origin

We identified nine articles indicating weaning failure owing to cardiovascular causes. The transition from invasive MV to spontaneous breathing places patients at risk for cardiovascular failure, especially if a history of cardiovascular disease is already present.^25^ Supplementary material S1 presents the retrieved papers highlighting the cardiovascular parameters identified using ultrasound that were associated with weaning failure.

The primary physiological changes during positive-pressure MV result from increased intrathoracic pressure, which affects pleural pressure (P_PL_) and transpulmonary pressure (P_TP_).^26^ An elevation in P_TP_ leads to a corresponding increases in right atrial pressure, thereby diminishing the pressure gradient for venous return and hindering blood flow to the right ventricle. This reduction ultimately decreases left ventricular preload, afterload, and cardiac output.^27^

During SBTs, these cardiopulmonary interactions exacerbate preload and afterload on the cardiac chambers, increasing the risk of myocardial ischaemia and weaning-induced pulmonary oedema (WIPO).^28^ In cases of mitral regurgitation, particularly with concurrent myocardial ischaemia, the likelihood of weaning failure is increased.^29^

After extubation, when spontaneous breathing is fully restored, both intrathoracic and right atrial pressures swing into negative values, enhancing venous return. However, as veins collapse during their transition from extrathoracic to intrathoracic compartments, extremely negative right atrial pressure may lead to flow limitations, as occurs with deep spontaneous inspiratory efforts or upper airway obstruction.^30^

The incidence of WIPO in patients undergoing SBT is approximately 60%.^31^ Factors such as heart disease, chronic obstructive pulmonary disease, and obesity have been identified as independent risk factors for developing WIPO.^32^ This condition is particularly evident during spontaneous breathing with significant negative swings in P_PL_ among patients with reduced left ventricular function. Consequently, it results in increased left ventricular afterload and right ventricular filling, promoting pulmonary oedema as a result of elevated pulmonary blood volume and capillary permeability.^30^ The gold standard for diagnosing WIPO is pulmonary artery occlusion pressure measurement; however, alternative noninvasive or minimally invasive methods have been proposed, including biochemical analysis using B-type natriuretic peptide, assessment of left ventricular filling pressure, evaluation of extravascular lung water, and, more recently, pulmonary ultrasound.^28^

Transthoracic echocardiography (TTE) can visualise both systolic and diastolic heart function to predict weaning outcomes.^8^ Systolic function is evaluated by measuring ejection fraction, whereas diastolic function is assessed using Doppler waves to analyse mitral inflow, where the early wave (E) reflects passive ventricular filling, and the late wave (A) indicates active atrial contraction. The parameters most associated with weaning failure from cardiovascular origin are higher ratio of early diastolic mitral inflow velocity to early diastolic septal mitral annulus velocity (mitral septal E/E′, higher diastolic dysfunction, left ventricular diastolic dysfunction, including increased left atrial pressure indicated by interatrial septal fixed rightward curvature). Failed extubation was associated with higher e/E′ values (odds ratio [OR] 1.27, 95% confidence interval [CI] 1.05–1.54), being 10.9 (interquartile range [IQR] 8.7–18.0) a cutoff point and higher left atrial area (OR 1.14, 95% CI 1.02–1.28), showed a cutoff point of 25.0 (IQR 19.3–32.0). Fixed rightward curvature of the interatrial septum showed a median of 7 for those patients who failed extubation (OR 12.95, 95% CI 2.73–61.41).^33^ Tissue Doppler imaging can also assess the mitral annulus in patients without mitral abnormalities, facilitating accurate evaluation of left ventricular relaxation (e’ wave) and left ventricular filling pressure (E/e’ ratio).^8^^,^^34^ Factors such as peak A wave <100 cm s^−1^, E/Ea >14, and inferior vena cava maximum diameter (IVCmax) >17 mm are associated with reintubation. In the weaning failure group, peak E-wave velocity was significantly higher (95 cm s^−1^ [75–115] *vs* 85 cm s^−1^ [61–100]; *P*<0.001), and E-wave deceleration time was shorter (128 ms [89–163] *vs* 147 ms [109–206]; *P*<0.001). An E velocity ≥0.6 m s^−1^ at baseline and E ≥1.2 m s^−1^ during SBT had 100% specificity and 15% sensitivity for predicting successful SBT, with a 91% sensitivity to identify failed SBT owing to WIPO.^35^

### Weaning failure from diaphragmatic dysfunction

Diaphragmatic dysfunction is characterised by a progressive decline in muscle strength, which can develop early during MV, termed ventilator-induced diaphragmatic dysfunction (VIDD), affecting up to 65% of patients in intensive care.^36^ Studies have shown that patients with brain injuries can experience significant atrophy of type I and type II fibres after only 18 h of controlled MV, with a reduction of more than 50% in diaphragm cross-sectional area.^37^ This disuse atrophy occurs not only in patients on controlled modes but also in assisted modalities if diaphragmatic work is not adequately monitored. A multicentre study involving 231 patients revealed that 50% of those on MV with pressure support experienced over-support, a primary mechanism of diaphragmatic atrophy as a result of disuse. Consequently, respiratory muscle weakness results from MV rather than the ventilatory mode.^38^ Another study found that diaphragmatic dysfunction is twice as prevalent as limb weakness, negatively impacting the weaning process.^39^

The significance of this issue is underscored by evidence that patients with MV-induced diaphragmatic dysfunction have poorer prognoses, increased extubation failure rates, and longer durations of MV,^40^ ultimately increasing mortality rates.^41^ The main risk factors for diaphragmatic dysfunction include the duration of MV and sepsis.^42^

Among noninvasive methods for evaluating diaphragmatic function, ultrasonography allows for assessment of muscle structure and function by examining thickness, excursion, and the ability to generate force through the diaphragmatic thickening fraction.^43^ A recent systematic review and meta-analysis found that both excursion and diaphragmatic thickening fraction have good specificity and sensitivity for predicting successful weaning, demonstrating good diagnostic accuracy.^44^ The presence of diaphragmatic dysfunction, evaluated by ultrasound, serves as a warning sign; during spontaneous ventilation tests with reduced support pressure and positive end-expiratory pressure (PEEP), it is crucial to assess diaphragmatic structure and function to ensure the diaphragm can handle the work of breathing post-extubation or to determine the need for noninvasive ventilatory support. The presence of diaphragmatic dysfunction evaluated by ultrasound should be considered as a warning sign as, when performing the spontaneous ventilation test and decreasing the supporting pressure and the PEEP, it is opportune to evaluate the diaphragmatic structure and function to ensure that the main engine of ventilation will be able to assume the work of breathing after extubation or decide on the need for noninvasive ventilatory support after the ventilatory support.^8^

**Fig 4:**
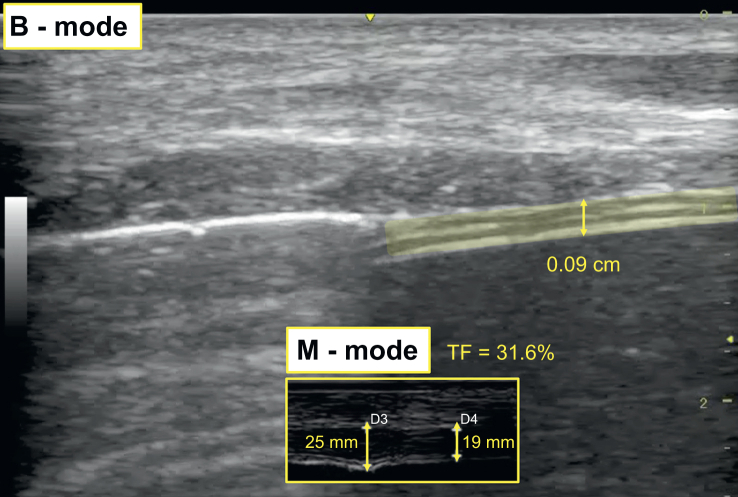
Diaphragmatic thickening. Ultrasonographic evaluation revealed diaphragmatic atrophy, observing a decrease in diaphragm thickness during expiration (0.09 cm; normal value: 0.13–0.15 cm), and a thickening fraction of 31.6%. Diaphragmatic thickening is seen with a 7.5–10 MHz linear probe at the level of the apposition zone. The end-expiratory thickness in expiration was 19 mm, whereas the end-inspiratory thickness in inspiration was 25 mm. The thickening fraction corresponded to: TF=(25–19)/19×100=31.6%.

Diaphragmatic dysfunction is identified by diaphragmatic excursion (DE), diaphragm thickening fraction (DTF), diaphragmatic rapid shallow breathing index (D-RSBI), and the ratio between VF and the ultrasonographic evaluation of diaphragmatic displacement (DD). Many studies reported cutoff points for DE, ≤1.1 cm being associated with weaning failure.^45^ Additionally, values of <20%,^46^ DE-RSBI  >1.38 bpm mm^−1^ and DTF-RSBI  >78.1 bpm %^−1^,^47^ were reported for weaning failure for DTF and D-RSBI, respectively. VF/DD ratio showed a sensitivity of 87.5 and specificity of 92.31 in predicting weaning outcomes,^48^ while VF/DTF of the right hemidiaphragm with a threshold value of ≤0.81 exhibited a sensitivity of 87.7% and specificity of 75% to predict extubation success (area under the receiver operating curve [AUROC] 0.762).^49^ Figure 4 shows VIDD after 10 days of controlled MV.

A recent study explored the association between WIPO, diaphragmatic dysfunction, and loss of aeration in patients facing difficult weaning. It found that WIPO was linked to diaphragmatic dysfunction, assessed through phrenic nerve stimulation, in 84% of patients who failed SBT.^31^ This suggests recruitment of auxiliary respiratory muscles in cases of diaphragmatic dysfunction. Furthermore, the lung ultrasound score (LUS) was significantly higher in patients with diaphragmatic dysfunction, indicating loss of aeration.

### Weaning failure from respiratory cause: loss of aeration, pulmonary oedema, and pleural effusion

**Fig 5:**
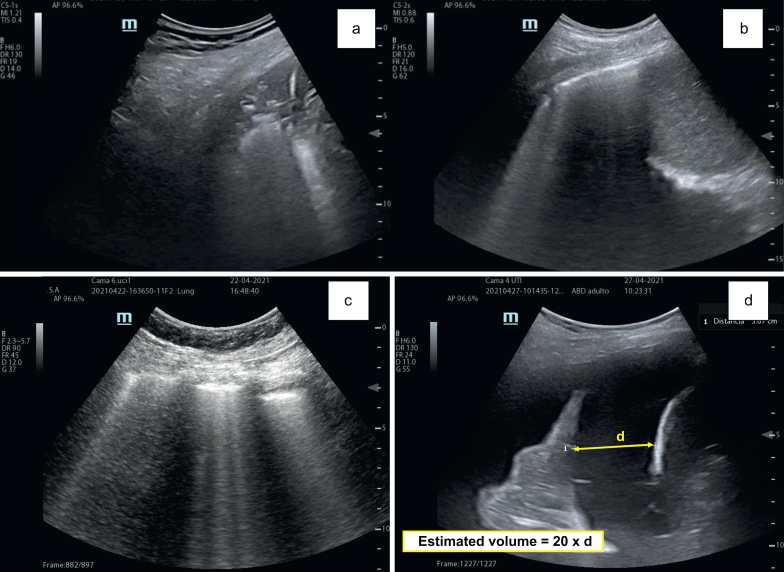
Consolidation and pleural effusion at lung ultrasound. The right lung base is seen with a convex probe. (a) Consolidation pattern with air bronchogram (indicated with arrow). (b) After 3 days, an improvement in lung ventilation is seen without consolidation pattern. (c) With convex probe in transverse plane at anterior level, B-lines of interstitial type secondary to weaning-induced pulmonary oedema (WIPO) are displayed. (d) With a convex probe in transverse position, perpendicular to the chest wall, at the level of the right base, extensive pleural effusion is seen. According to Balik's formula, a volume of 720 ml is estimated. Measuring the maximum separation between parietal and visceral pleural at maximal inspiration: 36 mm×20=720 ml.

Ultrasound has emerged as a preferred tool for diagnosing ARF, enabling easier identification of PEEP values and monitoring of MV.^50^^,^^51^ It is particularly effective for detecting pleural effusion, pneumonia, or pulmonary oedema, demonstrating greater sensitivity than radiographic techniques.^52^ The application of the BLUE protocol has enhanced diagnostic accuracy for the most common causes of respiratory failure, thanks to its high sensitivity and specificity. Given these advantages, ultrasound should be considered essential during the weaning process from MV and for monitoring clinical evolution in the post-extubation period, particularly in evaluating pulmonary aeration. Ultrasound can confirm the initiation of weaning in respiratory failure by verifying improvements in aeration compared with admission, indicating that the underlying cause necessitating MV is resolving. Figure 5a shows lung consolidation, and Figure 5b shows the resolution and aeration gain in the right lung base by ultrasound after 3 days of invasive MV.

Critically ill patients often present with consolidations or atelectasis, which can be differentiated by ultrasound based on the presence of dynamic or static air bronchogram patterns.^53^ A LUS above 17 points has been associated with weaning failure from impaired pulmonary aeration and can be applied during both the weaning process and the post-extubation period.^54^ Additionally, the presence of B-lines in anterior and lateral pulmonary fields, particularly when exceeding six, indicates pulmonary oedema induced by respiratory effort during spontaneous ventilation testing and weaning.^26^

After extubation, the absence of positive pressure contributes to increased venous return, allowing ultrasound to assess extravascular pulmonary water content. Previous studies in experimental models have shown that ultrasound can be more accurate than CT for this purpose, suggesting applicability to patients with diastolic failure or prolonged relaxation periods.^55^ Consequently, lung ultrasound can effectively distinguish increases in extravascular water content and differentiate lung disorders with parenchymal involvement.

Lung ultrasound has proven to be an excellent tool for diagnosing WIPO. Its main advantages include real-time, noninvasive diagnostics with good reproducibility, achievable within few minutes by a well-trained operator.^11^^,^^28^ The presence of B-lines, indicative of interstitial syndromes, can be detected with lung ultrasound before the exacerbation of clinical signs and symptoms.^28^ An increase in B-lines suggests elevated lung density and extravascular lung water, conditions seen in WIPO cases^56^ as shown in Figure 5c.

Loss of aeration is represented with LUS, alveolo-interstitial syndrome (defined as increased B-line artifacts), and disappearance of the peripheral parenchymal lesion (defined as the presence of C-lines—hypoechoic subpleural focal images with or without pleural line gap and disappearance of dynamic air-bronchogram). An anteroposterior LUS score >7^20^ or a conventional LUS score >10^21^ was associated with weaning failure; nevertheless, a value of >17 was reported as a cutoff point for weaning failure.^22^

A study involving 62 patients who underwent T-tube SBT found that 17 out of 33 patients who failed SBT exhibited WIPO, yielding a sensitivity and specificity of 88% and an AUROC of 0.91. The authors concluded that an increase of six B-lines across four lung fields accurately indicates WIPO.^28^ Another study confirmed the reliability of lung ultrasound in diagnosing WIPO, showing that in a cohort of 52 patients, 12 progressed to WIPO, representing 48% of those who failed. An increase of six or more B-lines yielded a sensitivity of 83.3% and a specificity of 82.5%, with an AUROC of 0.825.^11^ Thus, lung ultrasound appears to be a dependable method for identifying weaning failure caused by pulmonary oedema.

Patients with moderate to extensive pleural effusions at the onset of the spontaneous ventilation test are less likely to achieve successful extubation and tend to have longer hospital stays.^57^ Moderate to large pleural effusions are defined as a maximal interpleural distance  ≥15 mm (predicting an effusion volume of ≥300 ml); a large pleural effusion is defined by a maximal interpleural distance  ≥25 mm. Pleural effusions are common in critically ill patients, with an incidence of up to 62% in medical ICUs and a 41% incidence upon admission. Ultrasound can detect minor effusions often missed by portable radiography.^58^^,^^59^ Haga clic o pulse aquí para escribir texto. Moreover, some alveolar condensations can mimic pleural effusions on radiography, highlighting the utility of ultrasound for distinguishing between these conditions.^60^ The sensitivity and specificity of ultrasound, when benchmarked against chest CT as the gold standard, is 93%, providing the advantage of avoiding radiation while allowing bedside evaluation. Ultrasound also facilitates assessment of effusion volume, nature, and guides appropriate thoracentesis location. Complex or diffusely echogenic effusions are indicative of exudates, often corresponding to haemothorax or empyema, while hyperechogenic internal elements and fibrin septa can be observed in septate effusions.^61^

In mechanically ventilated patients, various methods can estimate pleural fluid volume, with the Balik formula being particularly practical. This formula allows estimation of the minimum amount of effusion present by measuring the maximum distance in millimetres between the visceral and parietal pleura during expiration and multiplying by 20, resulting in a significant correlation (r=0.72), as reported in Figure 5d.

The success rate for fluid extraction under ultrasound guidance stands at 100%, with no reported incidents of pneumothorax or bleeding.^62^ Thus, considering the efficiency, safety, and ease of ultrasound evaluation at the bedside, an ultrasound examination should be performed in the posterior basal pleuropulmonary regions to investigate pleural effusions that may lead to loss of pulmonary aeration and increased respiratory effort post-extubation.

### Weaning failure from airway obstruction

Sonographic evaluation of airway anatomy has been validated for several years, proving beneficial in critically ill patients. Applications include identifying predictors of difficult airway, labelling the cricothyroid membrane, selecting appropriate endotracheal tube sizes, confirming orotracheal intubation, assisting in tracheostomy procedures, and evaluating post-extubation laryngeal stridor and vocal cord mobility.^63^^,^^64^ Evaluation at the level of the vocal cords is referred to in the literature as transcutaneous laryngeal ultrasonography and is well-documented. Ultrasound can help predict the potential for post-extubation laryngeal oedema before extubation and subsequently evaluate vocal cord mobility in patients who develop stridor or dysphonia after extubation, providing a rapid, safe, and noninvasive method of assessment.

By utilising a high-frequency probe at the level of the thyroid cartilage, clinicians can visualise the air column in the centre of the vocal cords in intubated patients while keeping the cuff inflated. This allows for anatomical and functional evaluation during cuff deflation, helping to predict the likelihood of post-extubation stridor. A study conducted in 2006^18^ demonstrated that laryngeal tracheal ultrasonography (LTUS) could reliably assess vocal cords, laryngeal morphology, and airflow through the vocal cords or subglottic area in the presence of laryngeal oedema. During cuff inflation, ultrasound visualisation reveals a square-shaped air column, whereas deflation, in the absence of oedema, shows a trapezoidal shape with an increased measured distance. Measuring the air column width (ACW), which is the width of the acoustic shadow at the level of the cords before and after cuff deflation in the intubated patients, can predict the risk of post-extubation stridor. ACWD is defined as difference in the air column measurement in the intubated and deflated state. A difference of less than 0.8 mm is reported to be correlated with the development of post-extubation stridor. This study indicated that an ACW of 4.5 mm during cuff inflation correlated with a 7–8% post-extubation stridor rate, whereas a width greater than 6.4 mm indicated no stridor.^18^

**Fig 6:**
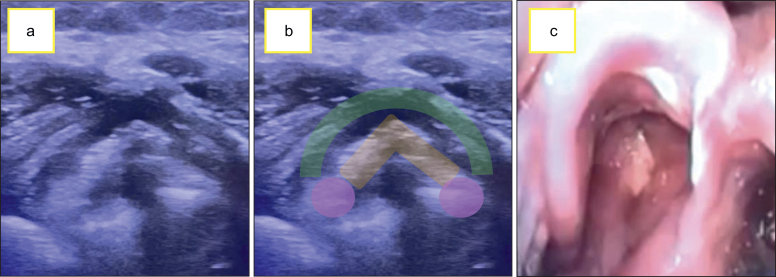
Airway obstruction as a result of laryngeal oedema. Ultrasound with linear probe, in transverse plane at the level of the thyroid cartilage. (a and b) A decrease in space between both vocal folds secondary to laryngeal oedema is seen. Green: thyroid cartilage; orange: vocal folds; purple: arytenoid cartilage. (c) Real image through video laryngoscopy during reintubation where the laryngeal oedema is seen.

Ultrasound has also been used to assess vocal cord mobility, particularly in cases of superior or recurrent laryngeal nerve lesions after neck surgeries.^65^ A systematic review recently reported that LTUS has a high sensitivity of 95% and specificity of 99% for detecting vocal cord immobility in adults, which is particularly valuable when access to endoscopic evaluation is limited or delayed.^66^ The ability of ultrasound to assess vocal fold conditions quickly and dynamically post-extubation, coupled with its capacity to visualise oedema, aids in managing stridor or post-extubation dysphonia. Figure 6 shows the LTUS of a patient who presented with laryngeal stridor and post-extubation dysphonia, finding that there was bilateral cord mobility but with laryngeal oedema, which initially promoted the local management of oedema.

### Limitations

This review has several limitations. First, there is significant heterogeneity among the studies included, as they vary in design, patient populations, and ultrasound techniques. The scoping review design was chosen because of the heterogenous outcomes and measurement techniques reported in the literature, making a systematic review and meta-analysis impractical. This heterogeneity can also make it difficult to draw strong conclusions that apply across different clinical settings. Furthermore, the findings may have limited generalisability and may not reflect the broader patient characteristics encountered in diverse healthcare environments. There is also the potential for bias, especially in observational studies, where selection and reporting bias can influence the reliability of the results. Additionally, the variability in ultrasound techniques used by different practitioners can lead to inconsistent results, complicating the synthesis of findings. Further, skilled personnel can be unavailable at the time of weaning readiness assessment, limiting the integration of ultrasonography into the weaning process. It is also worth noting that ultrasonography has several limitations, including inter- and intra-operator variability, need for continuous training, and possible difficult performance in certain patient conditions such as obesity, subcutaneous emphysema, or difficult ultrasonographic windows. Lastly, the absence of standardised protocols for utilising ultrasound parameters to predict weaning failure means that there can be significant differences in clinical practice, further challenging the applicability of the review's conclusions.

### Conclusions

Weaning accounts for >40% of patients' time on MV, making proper evaluation crucial for improving outcomes. This can reduce ICU stays and prevent post-extubation ventilatory failure that may lead to reintubation, increasing the risk of mortality. Ultrasound is a useful adjunctive tool for rapid and precise evaluation of critical illness and guiding decision-making. Its advantages include the ability to perform assessments at the bedside and dynamically visualise structures and functions noninvasively, providing a thorough understanding of diseases. Ultrasound helps assess pleural effusions, lung aeration before and after SBT, and the increase in extravascular pulmonary water owing to the loss of positive pressure. From a diaphragmatic perspective, ultrasound can identify dysfunction caused by MV, especially in patients with risk factors such as neuromuscular block, cardiovascular dysfunction, or ICU-acquired weakness. Additionally, it can be useful for evaluating the airway, including the air column and vocal cords (with inflated and deflated cuffs), to predict laryngeal oedema after extubation, and for assessing vocal cord mobility. In summary, ultrasound represents a valuable tool for optimising the weaning process, as it enables precise assessment of lung function and diaphragmatic performance, underscoring the need for its implementation in intensive care setting.

## Authors' contribution

Conception and design: all authors

Interpretation of literature: all authors

Revision for critical important intellectual content: all authors

Editing and approval of the version submitted: all authors

Writing first draft: PS, BS, AG, NP, DB

Editing first draft: RA, DB

Supervision: DB

## Declarations of interest

DB is a lecturer for Estor® and a member of the associate editorial board of *British Journal of Anaesthesia*. The other authors declare that they have no conflicts of interest.
