# Supplementary material for: Weaning failure from mechanical ventilation: a scoping review of the utility of ultrasonography in the weaning process
Source: Br J Anaesth. 2025 Mar 27;135(5):1441–55. doi: 10.1016/j.bja.2025.02.024 (PMC12597380; doi:10.1016/j.bja.2025.02.024)
Supplement: Multimedia component 1 [file mmc1.docx]

**Supplementary Material**

**Item S1. Observational studies included for review**

| First author | Year | Country | Study type | N. patients  included | N. patients  weaning failure | Weaning outcomes using ultrasound measurements |
| --- | --- | --- | --- | --- | --- | --- |
| Song J ^1^ | 2024 | China | single-center, prospective, observational | 51 | 15 | The study comprised 51 patients, 15 of whom reported weaning failure. During the SBT, the failure group had higher global, anterior, and antero-lateral LUS scores than the success group. According to receiver operating characteristic curve analysis, the areas under the curves for diaphragm thickening fraction (DTF), global and antero-lateral LUS scores during the SBT to indicate weaning failure were 0.678, 0.719, and 0.721, respectively. There was no relationship between the LUS scores and the average E/e' ratio during the SBT. A multivariate study found that antero-lateral **LUS score > 7 and DTF < 31%** during the SBT were independent predictors of weaning failure. |
| Wang Y ^2^ | 2024 | China | single-center, prospective, observational | 35 | 12 | The average age of the 35 patients in the research was (75 ± 9) years, and 12 (37%) were unable to be weaned from mechanical breathing. The lung ultrasonography score accurately predicted these failures with an AUC of 0.885 (95% CI 0.770-0.999, p < 0.001), with a threshold score of 10 providing 72.7% specificity and 92.3% sensitivity. AUCs were lower for previously known predictors of weaning failure, and there was no significant difference in right hemidiaphragmatic excursion between the groups. |
| Li C ^3^ | 2024 | China | single-center, prospective, observational | 35 | 18 | They analyzed 581 SWE and B-mode ultrasound films, 466 from the control group of 179 normal people and 115 from the patient group of 35 mechanically ventilated subjects in the intensive care unit (ICU). Seventeen of the patients successfully weaned, while eighteen failed. U-Net's deep neural network was used to automatically segregate diaphragm areas in dual-modal SWE and B-mode movies. After extracting high-throughput radiomics features, the statistical test and the least absolute shrinkage and selection operator (LASSO) were used to reduce feature dimension. The support vector machine (SVM) was used to determine the best classification models for both tasks. The right and left diaphragmatic excursion (LDE and RDE) decreased significantly in the failure group (P<0.001). Right expiratory thickening (RET) (cm) **(0.28 (0.27–0.33) vs. 0.27 (0.25–0.28), P=0.003), and** **right inspiratory thickening (RIT) (cm) (0.20 (0.18–0.21) vs. 0.18 (0.17–0.19), P=0.002), were signifcantly higher in the successful group than the failure group**. |
| Lin H ^4^ | 2024 | China | single-center, prospective, observational | 79 | 22 | DTF's sensitivity, specificity, and optimum cutoff value for effective weaning were 0.772, 0.727, and 0.293, respectively, with an area under the curve (AUC) of 0.72 (95%CI 0.59-0.86, p = 0.003). The sensitivity, specificity, and optimum cutoff value for IWI to predict effective weaning were 0.614, 0.909, and 53.00, respectively, with an AUC of 0.82 (95%CI 0.72-0.91, p < 0.001). The combination of DTF and IWI predicted successful weaning with sensitivity, specificity, and optimum cutoff values of 0.614, 0.909, and 17.848, respectively, and an AUC of 0.84 (95%CI 0.75-0.93, p < 0.001). |
| Fritsch SJ ^5^ | 2024 | Germany | single-center, prospective, observational | 50 | 12 | Tdi increased during spontaneous breathing [begin 3.41 (0.99) vs. end 3.43 (1.31) mm; P=0.01] and diaphragmatic excursion [begin 0.7 (0.8) vs. end 0.9 (0.6) cm; P=0.01]. The presence of positive end-expiratory pressure (PEEP) and pressure support did not influence the sonographic parameters significantly. The M. quadriceps femoris, in contrast, decreased slightly but constantly over the time [lower third: begin 1.36 (0.48) vs. end 1.28 (0.36) cm; P=0.054]. |
| Rajpal M ^6^ | 2024 | India | single-center, prospective, observational | 54 | 34 | 150 mechanically ventilated ICU patients were prospectively monitored over 18 months, with demographic and clinical data recorded. Extravascular lung water was measured by LUS in four intercostal spaces (ICS) from day 1 to day 5, day 7, day 10, and regularly afterward. The pulmonary fluid load was categorized as low (1-10), moderate (11-20), and high (21-32). Weaning result, length, mechanical breathing, ICU stay, and death were compared between individuals with and without EVLW. Out of 150 patients, 54 (36%) developed EVLW. The average lung score for our patients was 8.57 ± 6.0. The mean time for detecting EVLW was 1.43 ± 2.24 days. The lung score was low in 40 patients (26.67%), moderate in 9 patients (6.00%), and high in 5 patients (3.33%). Patients with EVLW had substantially higher rates of weaning failure (p=0.006), length of weaning, mechanical ventilation, ICU stay (p<0.0001), and overall death. |
| Sabetian G ^7^ | 2024 | Iran | single-center, prospective, observational | 50 | 24 | The median injury severity score was 75 (range: 42-75). **The failure group showed considerably lower right diaphragmatic excursion (DE) than the success group (P = 0.006).** Furthermore, the failure group showed a substantial reduction in both right and left DE from admission until the first weaning attempt from MV (P < 0.001). Both groups demonstrated a substantial reduction in inspiratory and expiratory thickness on both sides after weaning from MV compared to admission time (P < 0.001). The ROC analysis results showed that the Rapid shallow breathing index (RSBI) (Sensitivity = 91.67, Specificity = 100), respiratory rate (RR)/DE (Right: Sensitivity = 87.5, Specificity = 92.31), and RR/TF (Thickening Fraction) (Right: Sensitivity = 83.33, Specificity = 80.77) all had high sensitivity and specificity in predicting weaning outcomes. |
| Xin S ^8^ | 2024 | China | single-center, prospective, observational | 89 | 28 | The successful weaning group had 61 participants, whereas the unsuccessful weaning group had 28. The diaphragm contraction and relaxation rates were substantially greater in the failed weaning group compared to the successful weaning group (P < 0.05). The AUC for diaphragmatic peak contraction velocity, peak relaxation velocity, diaphragmatic contraction acceleration, and diaphragmatic relaxation acceleration was 0.81 (0.72-0.91), 0.85 (0.77-0.93), 0.74 (0.63-0.86), and 0.86 (0.78-0.94), respectively. |
| Boscolo A ^9^ | 2024 | Italy | single-center, prospective, observational | 44 | 16 | A total of 44 participants were recruited. Diaphragm dysfunction was seen in 14 participants (32%), all of them experienced difficulty weaning (78% of the subgroup of 18 patients). Difficult weaning resulted with **decreased diaphragm thickening (24 [20 to 29] vs. 39 [35 to 45]%) and neuroventilatory efficiency (34 [26 to 45] vs. 55 [43 to 62] ml/µV)** (both P < 0.001). The areas under the receiver operator curve indicating difficult weaning were 0.88 (95% CI, 0.73–0.99) for diaphragm thickening fraction and 0.85 (95% CI, 0.71–0.95) for neuroventilatory efficiency. The length of ventilation had a linear inverse relationship with both diaphragm thickness fraction and neuroventilatory efficiency. |
| Hyun J ^10^ | 2024 | Korea | single-center, prospective, observational | 146 | 11 | A total of 146 consecutive patients (mean age 70 years, 65.8% male) were enrolled. The median number of B-lines was 10 and linked with NT-pro-BNP levels (r2 = 0.132, p < 0.001). Early weaning failure was considerably greater in the positive LUS group (9 out of 64, 14.1%) than in the negative LUS group (2 out of 82, 2.4%) (p = 0.011). The positive LUS group had a higher risk of total re-intubation throughout the hospital stay (p = 0.004), duration of intensive care unit stay (p = 0.004), and length of hospital stay (p = 0.010). Positive LUS had a negative predictive value (NPV) of 97.6% for the main outcome. |
| Pu H ^11^ | 2023 | China | multicenter, prospective, observational | 126 | 6 | 51 of 126 patients lost diaphragmatic function at some point during mechanical breathing. Patients with diaphragmatic dysfunction were significantly more likely to develop new-onset sepsis (absolute risk difference 12.2%, 95% CI 1.4 to 23.0%, P=0.026) or ventilator-associated pneumonia (absolute risk difference 12.9%, 95% CI 1.4 to 24.4%, P=0.028), as well as die before hospital discharge (absolute risk difference 18.1%, 95% CI 3.7 to 32.5%, P=0.014). Furthermore, they spent more time on the ventilator (mean difference+1.3 days, 95% CI 0.1 to 2.5 days, P=0.035), were more likely to have a tracheotomy (absolute risk difference+11.0%, 95% CI 1.0 to 21.0%, P=0.030), and had a higher risk of extubation failure (absolute risk difference 8.5%, 95% CI 0.4 to 16.6%, P=0.039). Overall, they also had a significantly longer ICU stay (mean difference1.8 days, 95% CI 0.1 to 3.5 days, P=0.037) and a significantly longer hospital stay (mean difference 1.2 days, 95% CI 0.04 to 2.4 days, P=0.041) |
| He G ^12^ | 2023 | China | single-center, prospective, observational | 56 | 13 | Among individuals with a diaphragm thickness of > 2 mm, the parasternal intercostal muscle thickness percentage was 10.34 (IQR 5.41-16.00) and 6.04 (IQR 16.88-45.30) for the successful and unsuccessful groups. In patients with a diaphragm thickness of less than 2 mm, the parasternal intercostal muscle thickness fraction was 12.31 (IQR 9.14-19.81) and 13.79 (IQR 10.64-25.00) for the successful and failed groups, respectively (P =.346). The p0.1 values were 1.50 (IQR 0.85-2.90) and 4.20 (1.50-7.60), respectively (P =.072). The AUC values for predicting weaning failure with PIMTF, airway occlusion pressure at 100 msec (P0.1), and combined diagnosis were 0.721 (P =.017), 0.792 (P =.002), and 0.869 (P <.001), respectively.  The optimum threshold for predicting weaning failure result was **PIMTF ≥ 13.15 %**, with a sensitivity of 76.9%, specificity of 62.8%, positive predictive value of 38.46%, negative predictive value of 90%, positive likelihood ratio of 2.07, negative likelihood ratio of 0.37, and accuracy of 66.07%. The **combination of PIMTF > 13.15% and** **P0.1 ≥ 3.9 cmH2O** exhibited a sensitivity of 84.6%, specificity of 90.7%, positive predictive value of 76.92%, negative predictive value of 95.355%, positive likelihood ratio of 12.09, negative likelihood ratio of 0.13, and accuracy of 91.07%. |
| Huang D ^13^ | 2023 | China | multicenter, prospective, observational | 88 | 38 | The mean automatic diaphragmatic excursion was significantly lower among patients of the weaning-failure group, as compared with the weaning-success group (1.1 vs. 1.5 cm, p = 0.0163). Diaphragmatic velocity of excursion (cm/s) were similar among patients of both group (1.0 vs. 0.9, p = 0.2437). Mean diaphragmatic excursion via manual imaging assessment was 0.9 ± 0.4 cm in the weaning-success group and 0.9 ± 0.7 cm in the weaning-failure group (p = 0.6681). The mean diaphragmatic velocity of excursion via manual imaging assessment was 1.4 ± 0.7 cm/s in the weaning-success group compared with 1.4 ± 0.8 in the weaning-failure group, with no significant differences between groups. |
| Varón-Vega F ^14^ | 2023 | Colombia | multicenter, prospective, observational | 367 | 31 | They selected 367 participants who were receiving invasive mechanical ventilation and performed 456 SBTs on them, with a success rate of 76.5%. To forecast the success of the SBT, they developed the following equation: (0.56 x Cough) - (0.13 x DCV) + 0.25. The cutoff criterion of ≥0.83 resulted in 91.5% sensitivity, 22.1% specificity, and 76.2% total accuracy. The AUC-ROC value was 0.63. To estimate extubation success, they used the equation (5.7 × SBT) + (0.75 × Cough) - (0.25 × DCV) - 4.5. **The cutoff threshold of ≥ 1.25** resulted in 96.8% sensitivity, 78.4% specificity, and an overall accuracy of 91.5%. The AUC-ROC for this model was 0.91. |
| Ramaswamy A ^15^ | 2023 | India | single-center, prospective, observational | 60 | 12 | Of the 81 patients evaluated, 60 matched the inclusion criteria, with 49 undergoing successful SBT and 48 being extubated. A **parasternal intercostal muscle thickness fraction (PMTF)% cut-off value of more than or equal to 15.38%** was linked with the highest sensitivity (75%) and specificity (87.8%) in predicting extubation failure. |
| Laguado-Nieto M ^16^ | 2023 | Colombia | single-center, prospective, observational | 61 | 10 | The prevalence of diaphragmatic dysfunction (as determined by excursion and TFdi) was 40.98%. The sensitivity, specificity, positive predictive value, and negative predictive value for TFdi<20% were 86%, 24%, 75%, and 40%, respectively, with an area under the receiver operating characteristic (ROC) curve of 0.6. The ultrasonographic examination of diaphragm excursion, inspiratory and expiratory thickness, and TFdi (>20%) can predict success or failure of extubation with an area under the ROC curve of 0.87. A TFdi of >20% (OR 2.73) and >30% (OR 3.6) resulted with the best likelihood of successful extubation. However, with a cut-off of 35%, TFdi only had an OR of 1.97. |
| Jouseellin V ^17^ | 2023 | France | single-center, prospective, observational | 40 | 12 | The study comprised 40 patients, 12 (30%) of whom failed extubation. Before extubation, patients who failed extubation had a larger LUS (19 vs 10, p = 0.003) and a lower area accessible for breathing (352 vs 406 pixels, p = 0.042). Following extubation, the GI index and LUS were greater in the extubation failure group, whereas the surface accessible for ventilation was reduced. The RVD and CoV did not differ across groups. |
| Rajbanshi LK ^18^ | 2023 | India | single-center, prospective, observational | 113 | 16 | SBT and extubation were successful in 73 (71.6%) and 57 (55.8%), respectively. The AUC for lung ultrasonography in predicting successful SBT at the beginning and 30 minutes of SBT were 0.781 (CI 95% 0.674-0.888, p < 0.001) and 0.841 (CI 95% 0.742-0.941, p < 0.001), with cut-off values of 17.5 and 19.5, respectively. The AUC for LUS compared to tracheal extubation was 0.786 (CI 95% 0.694-0.879, p < 0.001) and 0.841 (CI 95% 0.756-0.925, p < 0.001) at 0 and 30 minutes, respectively. 57.5% of patients with A profiles tolerated effective SBT, while 48.3% of patients with C profiles failed SBT (p < 0.001). COPD, LUS, greater SOFA score, and longer time of mechanical ventilation all demonstrated a statistically significant unfavorable relationship with effective SBT. |
| Raj I ^19^ | 2023 | India | single-center, prospective, observational | 41 | 4 | They found that 68.29 (%) of patients with normal dTF and DE values and 21.95 (%) with somewhat lower dTF and DE values were successfully extubated, whereas 7.31 (%) with normal dTF and DE values were reintubated and 2.43 (%) were extubated onto non-invasive ventilation (NIV). |
| Hirolli D ^20^ | 2023 | India | single-center, prospective, observational | 43 | 15 | Of the 193 patients examined, 43 were enrolled, with 15 (20.9%) experiencing extubation failure. Patients who experienced extubation failure had higher RIS-i scores (p < 0.001) and lower VISAGE scores (p = 0.043). C-PEFR and diaphragm function (excursions and contraction velocity during coughing) were decreased in patients with extubation failure, although not statistically significant. Variables with p < 0.2 in univariate analysis (RIS-i, VISAGE, and diaphragm cough velocity) were analyzed using multivariate regression. The RIS-I score remained an independent predictor (odds ratio 3.691, 95% confidence range 1.5-8.67, p = 0.004). In a receiver operating characteristic analysis, RIS-i has an area under the curve of 0.963. A RIS-i score of 2 or above exhibited 94% specificity and 89% sensitivity in predicting extubation failure. |
| Eksombatchai D ^21^ | 2023 | Tailandia | single-center, prospective, observational | 130 | 8 | The mean diaphragmatic excursion of the right and left hemidiaphragm was 1.6 ± 0.7 cm and 1.5 ± 0.7 cm, respectively (p = 0.15). The mean diaphragmatic thickening fraction of the right and left hemidiaphragms was 41.1 ± 17.6% and 42.6 ± 18.4%, respectively (p = 0.25). The right hemidiaphragm had a substantially greater mean TPIA than the left (1.2 ± 0.4 s vs. 1.1 ± 0.4 s, p < 0.001). The RR/DTF from both hemidiaphragms was considerably lower in the successful extubation group compared to the failure group. In all groups, the RR/DTF of the right hemidiaphragm was 0.47 (0.33-0.64) vs 1.1 (0.6-2.32), p < 0.001, whereas the left hemidiaphragm was 0.45 (0.31-0.65) vs 0.78 (0.48-1.75), p < 0.001. Bilateral RR/DE was considerably lower in the extubation success group compared to the extubation failure group. Extubation success was associated with considerably larger bilateral diaphragmatic thickness percentage and right diaphragmatic excursion compared to failure. RSBI was lower in the successful extubation group compared to the failure group (53.1 ± 24 vs. 80.6 ± 52.2, p = 0.005). In comparison to the other metrics, the right RR/DTF had the best sensitivity and specificity. The RR/DTF of the right hemidiaphragm with a threshold value of ≤ 0.81 exhibited a sensitivity of 87.7% and specificity of 75% to predict extubation success (AUROC = 0.762). |
| Er B ^22^ | 2023 | Turkey | single-center, retrospective, observational | 32 | 18 | 32 patients were included. The average age (SD) was 79.3 ± 7.9 years, with 18 (56.3%) patients categorized as weaning failure. Diaphragmatic excursion during SBT was the sole statistically significant factor linked to weaning failure [2.37 cm (0.67) vs 1.43 cm (0.15), p= 0.0359]. There was no statistically significant difference in RSBI between the two groups [70.5 (46) vs 127.5 (80), p = 0.09]. Frailty was modestly linked with baseline diaphragm thickness and SBT excursion. |
| Al Tayar AS ^23^ | 2022 | Arabia Saudi | single-center, prospective, observational | 24 | 8 | US examination of the diaphragm and assessment of diaphragmatic excursion (DE) and diaphragm thickening fraction (DTF) were recorded 30 min after initiation of SBT.  DTF predicted weaning failure with area under the curve 0.96 and P value < .001 with sensitivity 100% and specificity 94% for the cutoff value ≤ 15%. DE for the cutoff ≤ 1.56 cm showed sensitivity 75% and specificity 69%, whereas ΔEAdi with cutoff value ≤ 4 μV showed sensitivity 25% and specificity 100%. Neuroventilatory efficiency with cutoff value ≤ 29 mL/μV showed sensitivity 50% and specificity 81%. All 3 parameters showed nonsignificant results with area under the curve 0.73, 0.56, and 0.62 and P values .08, .65, and .34, respectively. |
| Alam MJ ^24^ | 2023 | Bangladesh | single-center, prospective, observational | 200 | 12 | This prospective, double-blind trial included 200 people who were weaned off the ventilator after being ventilated for more than a week owing to respiratory failure. 100 conventional weaning (group A) and 100 weaning using ultrasonography (group B). Group A had considerably more patients who developed worsening in consciousness, tachypnea, desaturation, tachycardia, and hypertension, as well as chest X-ray infiltration and re-ventilation, than group B. Furthermore, group A patients spent much longer in the ICU than group B patients. |
| Xu Q ^25^ | 2022 | China | multicenter, prospective, observational | 96 | 37 | Twenty-five healthy volunteers and twenty mechanically ventilated patients participated in Study A. Study B included 96 patients (59 of whom were successfully weaned). DLS had a linear connection with DTF (R2=0.73, p<0.0001) and DE (R2=0.61, p<0.0001). The areas under the ROC curves for DLS, diaphragmatic thickening fraction DTF, RSBI, and DE in predicting successful weaning were 0.794, 0.794, 0.723, and 0.728, respectively. The best cut-off value for predicting DLS weaning success was less than -21%, with 89.19% sensitivity and 64.41% specificity. |
| Song J ^26^ | 2022 | China | single-center, prospective, observational | 110 | 37 | Of the 110 patients investigated, 37 (33.6%) failed to wean. The area under the ROC (AUROC) curves for RSBI, DE-RSBI, and DTF-RSBI in predicting failure weaning were 0.639, 0.813, and 0.859, respectively. DE-RSBI and DTF-RSBI showed considerably higher AUROC curves compared to RSBI (P = 0.004 and P < 0.001, respectively). To indicate failure weaning, the optimum cut-off values were RSBI > 51.2 breaths/min/L, DE-RSBI > 1.38 breaths/min/mm, and DTF-RSBI > 78.1 breaths/min/%. |
| Saravanan R ^27^ | 2022 | India | single-center, prospective, observational | 200 | 29 | DLS showed a linear connection with both DTF (Pearson R2 = 0.73, P < 0.0001) and DE (Pearson R2 = 0.61, P < 0.0001). However, there was a weak or no connection between DLS and diaphragmatic thickness (DTee, R2 = 0.01, P = 0.3336; DTei, R2 = 0.07, P = 0.0071). Similarly, there was a weak or no connection between DTF and diaphragmatic thickness (DTee, R2 = 0.002, P = 0.647; DTei, R2 = 0.07, P = 0.0113). |
| Shamil PK ^28^ | 2022 | India | single-center, prospective, observational | 50 | 9 | 50 patients satisfied the inclusion criteria, with 45 undergoing successful SBT and 41 successfully extubated. As a result, 18% of the research sample failed to wean. D-RSBI and RSBI showed areas under the receiver operator characteristic (ROC) curves of 0.97 and 0.70, respectively (p < 0.0001). The Pearson correlation coefficient between RSBI and D-RSBI was 0.81 (p-value <0.001). |
| Lalwani LK ^29^ | 2022 | India | single-center, retrospective, observational | 104 | 37 | Of the 110 patients investigated, 37 (33.6%) failed to wean. The area under the ROC (AUROC) curves for RSBI, DE-RSBI, and DTF-RSBI in predicting failure weaning were 0.639, 0.813, and 0.859, respectively. DE-RSBI and DTF-RSBI showed considerably higher AUROC curves compared to RSBI (P = 0.004 and P < 0.001, respectively). To indicate failure weaning, the optimum cut-off values were RSBI > 51.2 breaths/min/L, DE-RSBI > 1.38 breaths/min/mm, and DTF-RSBI > 78.1 breaths/min/%. |
| Kundu R ^30^ | 2022 | India | multicenter, prospective, observational | 60 | 27 | The patients in Group S (success weaning) had a significantly lower LUS score both before and after SBT compared to Group F (failure weaning). The patients in Group F had a significantly higher change in LUS following SBT compared to Group S (P = 0.005). The patients in Group F had a lower DTF than those in Group S (P = 0.04). The patients in Group S showed a higher incremental VTI response to a passive leg raise compared to Group F (P < 0.001). For DTF, the optimal cut-off point for predicting weaning success was ≥ 26% (sensitivity 90.91%, specificity 37.04%, +veLR 1.4439, –veLR 0.2455). The optimal cut-off value for a VTI change to PLR was ≥ 10.2% (sensitivity 84.85%, specificity 66.67%, 2.5455, +veLR 0.2273, –veLR 0.51). In this model, DTF < 26% was a significant predictor of weaning failure in our study population with an odds ratio of 6.20 (95% CI: 1.06–36.04, P = 0.04). The percentage change in VTI to a PLR of less than 10.2% predicted weaning failure with an odds ratio of 6.16 (95% CI: 1.14–33.13, P = 0.03). |
| Amara V ^31^ | 2022 | India | single-center, prospective, observational | 81 | 65 | Expiratory muscle thickness followed the pattern of RA > IO > EO > TA in both simple and difficult/prolonged weaning groups. DE > 1.79 cm indicated simple weaning, and LUS was considerably decreased in individuals with simple weaning. |
| Vetrugno L ^32^ | 2022 | Italy | multicenter, prospective, observational | 57 | 25 | There were 57 patients enrolled, and 25 of them (44%) failed SBT. The median duration of invasive ventilation was 14 days (IQR 7-22). Within 24 hours of weaning, the median DTF was 28% (IQR 22-39%), as was the RASS score (-2 vs -2; p = 0.031), Kelly-Matthay score (2 vs 1; p = 0.002), and inspiratory oxygen fraction (0.45 vs 0.40; p = 0.033). Patients who failed weaning had a lower PaO2/FiO2 ratio (176 vs 241; p = 0.032), as well as a longer critical care stay (27 vs 16.5 days; p = 0.025). The generalized linear regression model did not include any factors that might predict weaning failures. DTF was linked with pH (RR 1.56 × 1027; p = 0.002); Kelly-Matthay score (RR 353; p < 0.001); RASS (RR 2.11; p = 0.003); PaO2/FiO2 ratio (RR 1.03; p = 0.05); SAPS2 (RR 0.71; p = 0.005); hospital and ICU duration of stay (RR 1.22 and 0.79, respectively; p < 0.001 and p = 0.004). |
| Bertoni M ^33^ | 2022 | Italy | single-center, prospective, observational | 73 | 11 | Fifty-seven COVID-19 patients (78%) had ICUAW, whereas 59 (81%) had DW. The two disorders coexisted in 48 patients (65%), however there was no correlation (χ2 = 1.06, p = 0.304). In the adjusted analysis, ICUAW was independently associated to VFDs at 28 days (estimate difference 6 days, p = 0.016) and WIND (OR of 3.62 for having WIND other than short weaning), but not DW. The linear mixed model found a significant but minor connection between MIP and TFdi (p < 0.001). |
| Pierrakos C ^34^ | 2021 | Belgium, Italy, Netherlands | multicenter, prospective, observational | 137 | 84 | This retrospective worldwide multicenter investigation included patients with COVID-19-related acute respiratory distress syndrome (ARDS) who had at least one LUS study within 5 days of starting invasive mechanical ventilation. Our study comprised 137 individuals with COVID-19-related acute respiratory distress syndrome. Independent of ARDS severity, the global LUS score was linked with effective liberation from mechanical ventilation (hazard ratio [HR]: 0.91, 95% confidence interval [CI] 0.87-0.96; P = 0.0007) but not with 28-day mortality (HR: 1.03; 95% CI 0.97-1.08; P = 0.36). The global LUS score's prognostic value was not increased by subpleural consolidation or pleural line abnormalities. Examinations within 24 hours of intubation revealed no prognostic value. |
| Li S ^35^ | 2021 | China | single-center, prospective, observational | 101 | 32 | Successful extubation resulted in a higher median DE (1.64 cm vs. 0.78 cm, p = 0.001). Patients with successful extubation had a higher DTF compared to those who failed (49.48% vs. 27.85%, p = 0.001). The areas under the receiver operating curves for the RSBI, LUS, DE, and DFT were 0.680, 0.764, 0.831, and 0.881. DTF≥ 30%, DE≥ 1.3 cm, LUS≤ 11, and RSBI≤ 102 were the most accurate predictors of successful weaning. The specificity of DTF (84%) in predicting weaning result was greater than that of RBSI (53%), LUS (55%), and DE (62%). The sensitivity of DTF (94%) was higher than that of RBSI (85%), LUS (71%), and DE (65%). The combination of RSBI, LUS, DE, and DTF had the greatest AUC (0.919), with 96% sensitivity and 89% specificity. |
| Helmy MA ^36^ | 2021 | Egypt | single-center, prospective, observational | 22 | 13 | Diaphragmatic excursion had the highest AUC for predicting effective weaning (right side: AUC [95% CI]: 0.996 [0.838-1.000], sensitivity: 100%, specificity: 92%, cut-off value: 11 mm; left side: AUC [95% CI]: 1.000 [0.846-1.000].The RSBI (AUC [95% CI]: 0.782 [0.557−0.927], sensitivity: 100%, specificity: 54%, cut-off value: < 97) and the P/F ratio (AUC [95% CI]: 0.774 [0.547−0.992], sensitivity: 78%, specificity: 77%, cut-off value: 281) had the highest sensitivity, specificity, and cut-off values. |
| Fossat G ^37^ | 2021 | France | single-center, prospective, observational | 100 | 18 | RSBI and RSBI/DE showed AUCs with 95% confidence intervals consistently extending below 0.50, either at the 5th (0.55 [0.36-0.74] and 0.55 [0.34-0.75], respectively) or at the 25th minute of SBT (0.49 [0.27-0.71] and 0.50 [0.29-0.68], respectively) for predicting weaning success at 72 h or at 7 days (5th min: 0.53 [0.37-0.70] and 0.54 [0.37-0.70], respectively; 25th min: 0.54 [0.37-0.71] and 0.55 [0.39-0.71], respectively). An exploratory index incorporating accessory respiratory muscle activity, DE, DTF, and respiratory rate at 5th min of SBT showed AUCs for predicting extubation success at 7 days in the 78 patients with DTF measurement (0.77 [0.64-0.90]) that were significantly higher than the RSBI (P = 0.017) and RSBI/DE (P < 0.001) in the same respective populations. |
| Dres M ^38^ | 2021 | France - Canada | multicenter, prospective, observational | 122 | 21 | Extubation failure occurred in 21 of 122 enrolled patients (17%). Patients with extubation failure had higher median (interquartile range (IQR)) Dyspnoea-VAS and IC-RDOS scores compared to those with success: 7 (4-9) against 3 (1-5) (p<0.001) and 3.7 (1.8-5.8) versus 1.7 (1.5-2.1) (p<0.001). Patients with extubation failure had a significantly higher median (IQR) ratio of parasternal intercostal muscle to diaphragm thickening fraction and lower MRC than those with extubation success: 0.9 (0.4-2.1) versus 0.3 (0.2-0.5) (p<0.001) and 45 (36-50) versus 52 (44-60) (p=0.012). The thickening percentage of the parasternal intercostal and its ratio to diaphragm thickening had the greatest area under the receiver operating characteristic curve (AUC) for early prediction of extubation failure (0.81). Dyspnoea-VAS and IC-RDOS had AUCs of 0.78 and 0.74, respectively. |
| Dres M ^39^ | 2021 | French | single-center, prospective, observational | 53 | 31 | Fifty-three patients were included, and 31/53 (58%) failed the spontaneous breathing challenge, including 24/31 (77%) with weaning-induced pulmonary oedema. Diaphragm dysfunction was found in 33/53 (62%) individuals. Diaphragm malfunction or weaning-induced pulmonary oedema occurred in 26/31 (84%) of the individuals who failed the spontaneous breathing exercise. Weaning-induced pulmonary oedema occurred in 20/33 (61%) individuals with diaphragm dysfunction vs 4/20 (20%) patients without (p = 0.005). |
| Cammarota G ^40^ | 2021 | Italy | single-center, prospective, observational | 100 | 21 | Extubation was effective in 79 cases, but failed in 21. Subjects who failed extubation had significantly higher median inspiratory peak excursion velocity (3.1 [IQR 2.0-4.3] vs 1.8 [1.3-2.6] cm/s, P <.001), mean velocity (1.6 [IQR 1.2-2.4] vs 1.1 [IQR 0.8-1.4] cm/s, P <.001), and acceleration (8.8 [IQR 5.0-17.8] vs 4.2 [IQR 2.4-8.0] cm/s2, P =.002) compared to those who were successfully extubated. Subjects who failed extubation had higher median expiratory peak relaxation velocity (2.6 [IQR 1.9-4.5] vs 1.8 [IQR 1.2-2.5] cm/s, P <.001), mean velocity (1.1 [IQR 0.7-1.7] vs 0.9 [IQR 0.6-1.0] cm/s, P =.002), and acceleration (11.2 [IQR 9.1-19.0] vs 7.1 [IQR 4.6-12.0] cm/s2, P =.004). |
| Trifi A ^41^ | 2021 | Tunis | single-center, prospective, observational | 30 | 16 | Compared to the SV group, patients in the MV group had a greater end expiratory diameter (2.09 ± 0.6 vs. 1.76 ± 0.32 mm, p=0.01) and a lower DTF (39.9 ± 12.5% vs. 49.0 ± 20.5%, p=0.043). Fourteen of the 30 ventilated patients were successfully weaned. There was no significant connection found between DTF and weaning duration (Rho=-0.464, p=0.09). A DTF value greater than 33% was nearly significantly linked with weaning success (OR = 2; 95% CI = [1.07-3.7], p = 0.05), with a sensitivity of 85.7%. |
| Er B ^42^ | 2021 | Turkey | single-center, prospective, observational | 38 | 15 | Thirty-eight patients were evaluated for weaning, 15 (39.4%) of whom failed. The median body mass index (BMI) was lower, while the median clinical frailty scale (CFS), vasopressor use, duration of mechanical ventilation, intensive care, and hospital mortality rate were higher in the weaning failure group, and the median TRF + VI (14.0 [12.3-26.2] vs 23.6 [21.3-27.1] mm, p = 0.03) and median DE (19.4 [14.6-24.0] vs 25.9 [19.3-38.5] mm, p = 0.045) were lower. The two groups had identical median Tdi values (1.9 [1.5-2.3] vs 2.0 [1.7-2.4] mm, p = 0.26). In ROC analysis, the area under the curve for TRF + VI was 0.71 (95% CI: 0.51-0.90; p = 0.035), and the 21 mm cut-off had a sensitivity of 82% and a specificity of 57%. After correcting for age, gender, BMI, and CFS, binary logistic regression analysis found that TRF + VI < 21 mm was the single predictor of weaning failure, with an odds ratio of 10.5 (95% CI: 1.1-97.8, p = 0.038). |
| Gok F ^43^ | 2021 | Turkey | single-center, prospective, observational | 62 | 22 | The research included sixty-two patients. The research population was primarily made up of trauma patients (77%). The cut-off value for RSBI was 64. The positive prediction value (PPV) for extubation success was 97%. The T-tube stage yielded cut-off values of 27.5 for DTF, 1.3 cm for DE, and 6.5 for LUS scores. The PPVs of all sonographic parameters were more than 90%. At the first stage, weaning and extubation failure rates were 35 and 9.6%, respectively. RSBI was discovered as a strong parameter in influencing extubation success (r=0.774, p≤0.001) and marginally linked with sonographic characteristics. |
| Whebell S ^44^ | 2020 | Australia | single-center, prospective, observational | 18 | 1 | During an SBT, ultrasonography measurements were performed on 18 participants. Four were unable to envision DTF. The DTF (n = 14, 32.41 ± 32.21 vs 23.19 ± 17.42, P =.33) and DE (n = 18, 1.72 ± 0.63 vs 1.66 ± 0.59, P =.63) did not vary significantly over time. Diaphragmatic contraction speed increased with time (n = 18, 2.21 ± 1.25 vs. 2.67 ± 1.61, P =.007). The diaphragmatic quick shallow breathing score worsened throughout time (n = 18, 1.65 ± 1.02 versus 2.08 ± 1.51, P =.03). There was no significant difference in the presence of DD. Diaphragmatic dysfunction caused by DTF 8/14 against 10/14, DE 4/18 compared 3/18, and DRSBI 7/18 versus 9/18. No patients failed SBT, although one patient failed extubation. |
| Porto D ^45^ | 2020 | Brazil | multicenter, prospective, observational | 86 | 15 | Extubation failure was reported in 15 (17.4%) patients. Two (5.7%) of the preload-dependent patients experienced extubation failure. Of the 51 preload-independent patients, 13 (25.5%) experienced extubation failure (P = 0.018). 86.7% of the 15 patients with extubation failure tested negative for PLR. The mean VTI fluctuation with the PLR maneuver was 3.7 ± 6.9% and 10.5 ± 11.5% in patients with and without extubation failure, respectively (P = 0.005). Preload independence was linked to the requirement for reintubation in the multivariable model (odds ratio, 5.3; 95% confidence range, 1.1-25.9; P = 0.038), even after controlling for sex, PaCO2, MV time before the SBT, and E/e′ ratio. |
| Elshazly MI ^46^ | 2020 | Egypt | single-center, prospective, observational | 62 | 28 | The successful extubation group showed a significant increase in diaphragmatic excursion and thickness fraction (p<0.001), as well as a negative correlation between diaphragmatic function and mechanical ventilation duration and Acute Physiology and Chronic Health Evaluation II. The diaphragmatic excursion cutoff value predictive of weaning was 1.25 cm, with a specificity of 82.1% and a sensitivity of 97.1%, respectively, while the diaphragmatic thickness cutoff value indicative of weaning was 21.5%, with a specificity of 60.7% and a sensitivity of 91.2%. |
| Fossé Q ^47^ | 2020 | France | single-center, prospective, observational | 25 | 13 | Thirty patients were recruited, and 930 respiratory cycles were examined. Twenty-five individuals were selected for the analysis. There was a strong association between ΔPdi and ΔSMdi (R = 0.45, 95% CIs [0.35 0.54], p < 0.001). Individual correlation is significant in 8 patients out of 25 (r = 0.55-0.86, all p < 0.05, vs r = -0.43-0.52, all p > 0.06). Changing ventilation conditions had comparable effects on ΔPdi and ΔSMdi. Patients with a non-significant ΔPdi-ΔSMdi connection had a higher respiratory rate than those with a significant ΔPdi-ΔSMdi association (median (Q1-Q3), 25 (18-33) vs. 21 (15-26) breaths.min-1, respectively). |
| Goudelin M ^48^ | 2020 | France | single-center, prospective, observational | 59 | 12 | Twelve of 59 patients failed SBT, and all developed WIPO. Successful SBT patients had decreased body weight (-2.5 kg [-4.8; -1] vs. +0.75 kg [-2.95; +5.57]: p = 0.02) and cumulative fluid balance (-2326 ml [-3715; +863] vs. +143 ml [-2654; +44]). Patients with WIPO experienced more significant central hemodynamic changes from SBT, including higher E wave velocity (122 cm/s [92; 159] vs. 93 cm/s [74; 109]: p = 0.017), higher E/A ratio (2.1 [1.2; 3.6] vs. 0.9 [0.8; 1.4]: p = 0.001), and shorter E wave deceleration time (85 ms [72; 125] vs. 147 ms [103; 175]: p = 0.004). Following echocardiography-guided therapy, all patients who failed the initial SBT were successfully extubated.  The fluid balance was negative (-2224 ml [-7056; +100] vs. +146 ml [-2654; +4434]: p = 0.005). SBT resulted in lower left ventricular filling pressures (E/E′: 7.3 [5; 10.4] vs. 8.9 [5.9; 13.1]: p = 0.028), as well as a smaller increase in E wave velocity (+ 10.6% [− 2.7/ + 18] vs. + 25.6% [+ 12.7/ + 49]: p = 0.037) and mitral regurgitation area. |
| Bouhemad B ^49^ | 2020 | France, Italy, Saudi Arabia | single-center, prospective, observational | 40 | 18 | Weaning or extubation failure was found in 45% (95% confidence interval 28-61) of patients. ROC analysis for the capacity of global SBT LUS to predict weaning/extubation failure and extubation failure yielded AUC values of 0.80 and 0.81, respectively. The AUC for anterolateral SBT LUS in predicting weaning/extubation failure was 0.79 and 0.81, respectively. LV filling pressure increased during SBT, but the anterolateral LUS score did not rise. In contrast, an increase in anterolateral LUS was found without increased filling pressure and was linked with extubation failure. There was no correlation between E/Ea and global or anterolateral SBT LUS. |
| Krishnakumar M ^50^ | 2020 | India | single-center, prospective, observational | 8 | 0 | A total of 18 patients were assessed for eligibility during a 5-month period, with eight meeting the inclusion criteria. There were 63 data points available for analysis across these eight participants. A successful breathing trial was indicated by a reduction in Edi (1.22 μV for every 30 min increase in weaning time; 0.69 μV for every day of weaning) and a rise in diaphragm excursion (2.81 mm for every 30 min increase in weaning duration; 2.18 mm for every day). |
| Xia J ^51^ | 2019 | China | single-center, prospective, observational | 49 | 6 | This was a prospective, single-center study. Lung ultrasound aeration score (LUS) and diaphragmatic thickening fraction (DTF) were assessed during silent breathing 1 hour before SBT (T-1), 30 minutes (T1), and 120 minutes (T2). The right and left DTF were compared between patients with LUS > 14 (high lung aeration loss), regarded at high risk of post-extubation discomfort, and those with LUS <14 (low lung aeration loss). They evaluated the connection between LUS and DTF, as well as changes from T-1 to T2 in individuals with LUS ≥14. Of 49 patients evaluated, 33 had LUS ≥ 14 and 16 had LUS < 14 at T1. Patients with LUS ≥14 had substantially greater DTF at T1 compared to those with LUS <14. The right median (IQR) DTF was 22.2% (17.1 to 30.9%) vs. 14.8% (10.2 to 27.0%) (p = 0.035), and the left median (IQR) DTF was 25.0% (18.4 to 35.0%) vs. 18.6% (9.7 to 24.2%) (p = 0.017), respectively. The LUS and DTF exhibited a moderate positive correlation (Rho = 0.3, p = 0.014). The LUS increased significantly from T-1 to T1, but remained same between T1 and T2. The DTF was steady from T1 to T2. |
| Zhang X ^52^ | 2019 | China | single-center, prospective, observational | 37 | 12 | Twenty-five patients successfully extubated, while 12 failed. The area under receiver operator characteristic curve (AUCROC) of DE30 and ΔDE30-5 (the variation between 30 and 5 min) were 0.762 and 0.835, respectively. A cutoff value of DE30 > 1.72 cm and ΔDE30-5 > 0.16 cm were associated with a successful extubation, with a sensitivity of 76% and 84%, and a specificity of 75% and 83.3%. The DE30 plus ∆DE30-5 predictive probability equation was P = 1/[1 + e-(-5.625+17.689×∆DE30-5+1.802×DE30)]. A cutoff value of P > 0.626 was related with a successful extubation, with an AUCROC of 0.867, a sensitivity of 92%, and a specificity of 83.3%. |
| Varón-Vega F ^53^ | 2019 | Colombia | single-center, prospective, observational | 84 | 17 | A total of 84 patients were involved, with 79.8% (n=67) successfully extubated and 20.2% (n=17) unsuccessfully extubated. Diaphragm contraction speed had the highest discriminating ability for predicting extubation success, with an AUC-ROC of 0.70 (p=0.008). |
| Eltrabili HH ^54^ | 2019 | Egypt | single-center, prospective, observational | 30 | 13 | Thirty individuals were included in the research; 17 were successfully extubated (56.6%), whereas 13 failed (43.4%). The time until the first liberation attempt was substantially shorter in the liberation-success group, 2.3 (0.7) days, than in the liberation-failure group, 5.8 (4.7) days; P =.02. The optimal cutoff value for predicting liberation success was ≥30.7%, with 94.1% sensitivity and 100% specificity. The area underneath the curve was 0.977. Diaphragmatic excursion of ≥10.4 mm yielded a 94% sensitivity and 85% specificity for predicting liberation success, with an area under the curve of 0.85. A quick shallow breathing index of ≤44 demonstrated 100% specificity and 76% sensitivity, with an AUC of 0.9. |
| Vivier E ^55^ | 2019 | France | multicenter, prospective, observational | 191 | 33 | Over a 20-month period, 191 at-risk patients were investigated. Of them, 33 (17%) were termed extubation failures. The proportion of patients with diaphragmatic dysfunction was similar between those who successfully extubated and those who failed to extubate: 46% versus 51% using excursion (P =.55) and 71% vs 68% using thickening (P =.73). The excursion and thickening values did not differ between the success and failure groups: excursion was 14 ± 7 mm vs 11 ± 8 (P =.13) and thickening was 29 ± 29% vs 38 ± 48% (P =.83). Extubation failure rates were 7%, 22%, and 46% for patients with effective, moderate, and ineffective coughs (P <.01). Ineffective coughing was the sole independent predictor of extubation failure. |
| Ferré A ^56^ | 2019 | France | single-center, prospective, observational | 42 | 33 | SBT failed in 33 patients. WIPO handled 17 lawsuits, all of which failed. The highest diagnosis accuracy was achieved with a Delta-B-lines ≥ 6. In 15 WIPO instances, the number of B-lines rose by ≥ 6 (13 cases had a rise of > 8 B-lines). Two out of 16 instances with SBT failure but no WIPO had Delta-B-lines of ≥6. Among the 33 cases of SBT failure, this profile detected WIPO with a sensitivity of 88% (64-98) and specificity of 88% (62-98) [area under the receiver operating characteristic curve 0.91 (0.75-0.98)]. Two of the 29 successful SBT patients had Delta-B-lines of ≥6. |
| González-Aguirre JE ^57^ | 2019 | Mexico | single-center, prospective, observational | 82 | 24 | Eighty-two patients were enrolled, with 24 (29.2%) failing to extubate. Univariate analysis revealed that DSF (Youden's J: >30% [sensibility and specificity 62 and 50%, respectively]) and the number of B-line regions (Youden's J: >1 zone [sensibility and specificity 66 and 92%, respectively]) were significantly associated with extubation failure (area under the curve 0.66 [0.52-0.80] and 0.81 [0.70-0.93], respectively). In binomial logistic regression, only the number of B-line areas is substantially associated to extubation failure (OR 5.91 [2.33-14.98], P<.001). |
| Tongyoo S ^58^ | 2019 | Thailand | single-center, prospective, observational | 52 | 14 | Fourteen out of 52 patients (mean age 65.9 ± 17.8 years) failed to wean. The primary causes of respiratory failure were severe pneumonia, metabolic acidosis, and septic shock. Reintubation was linked with BMI > 24, peak A wave < 100 cm/s, E/Ea > 14, and inferior vena cava maximum diameter (IVCmax) > 17 mm, according to a univariate analysis. Multivariate research found that E/Ea > 14 and IVCmax > 17 mm are independent predictors of weaning failure. |
| Rittayamai N ^59^ | 2019 | Thailand | single-center, prospective, observational | 45 | 13 | During SBT, TFditidal and RSBI levels considerably rose (TFditidal0 vs. TFditidal30 = 29.8 ± 13.8 vs. 37.4 ± 13.0%; p <.001, and RSBI0 vs. RSBI30 = 64.8 ± 25.9 vs.70.8 ± 29.1 breaths/min/L; p =.034). There was no significant difference in TFditidal between SBT failure (n = 13) and SBT success, except at the start of the trial (p =.043); nevertheless, RSBI rose considerably throughout SBT. No variations in TFdimax or DEmax were seen between the groups. |
| Haji K ^60^ | 2018 | Australia | single-center, prospective, observational | 53 | 11 | Fifty-three participants who had been intubated for more than 48 hours and were deemed ready for extubation underwent a 60-minute pre-extubation weaning trial (pressure support ≤ 10 cmH2O and positive end expiratory pressure 5 cmH2O). Prior to extubation, ultrasound was used to assess left ventricular ejection fraction, left atrial area, early diastolic trans-mitral flow velocity wave (E), early diastolic trans-mitral flow velocity wave/late diastolic trans-mitral flow velocity wave (E/A), early diastolic trans-mitral flow velocity wave/early diastolic mitral annulus velocity (E/E'), interatrial septal motion, lung loss of aeration score, and diaphragm movement. At the conclusion of the weaning experiment, the quick shallow breathing index and serum B-type natriuretic peptide levels were determined. Weaning success and failure were judged using predefined criteria. The decision to extubate was at the discretion of the treating intensivist. Failure of extubation was defined as re-intubation, noninvasive ventilation, or death within 48 hours after extubation. Eleven of the 53 extubated individuals failed to extubate. Failed extubation was associated with diabetes, ischaemic heart disease, higher E/E' (OR 1.27, 95% CI 1.05-1.54), left atrial area (OR 1.14, CI 1.02-1.28), fixed rightward curvature of the interatrial septum (OR 12.95, CI 2.73-61.41), and higher loss of aeration score of anterior and lateral regions of the lungs (OR 1.41, CI 1.01-1.82). |
| Abbas A ^61^ | 2018 | Egypt | single-center, prospective, observational | 50 | 13 | A total of 50 AECOPD patients who required mechanical breathing for more than 48 hours and were willing to undergo an SBT were included. Of them, 37 (74%) were successfully removed from mechanical ventilation. Among the 13 patients who failed the weaning trial, 8 (62%) failed the SBT and had to be reconnected to the ventilator, 2 (15%) were reintubated within 48 hours of extubation, and 3 (23%) required NIV assistance within 48 hours. D-RSBI and RSBI showed areas under the ROC curves of 0.97 (p<0.001) and 0.67 (p<0.06), respectively. |
| Palkar A ^62^ | 2018 | England | multicenter, prospective, observational | 73 | 20 | Of the 73 patients investigated, 20 did not succeed in extubation. During SBT, diaphragm excursion was 1.65 ± 0.82 and 2.1 ± 0.9 cm (P =.06), inspiratory duration was 0.89 ± 0.30 and 1.11 ± 0.39 s (P =.03), and E-T index was 1.64 ± 1.19 and 2.42 ± 1.55 cm-s (P <.03) in the "failure" and "success" groups, respectively. The mean difference in E-T index between A/C and SBT was -3.9 ± 57.8% in the failure group and 59.4 ± 74.6% in the success group (P <.01). A drop of less than 3.8% in diaphragmatic E-T index between A/C and SBT predicted successful extubation with a sensitivity of 79.2% and a specificity of 75%. |
| Razazi K ^63^ | 2018 | France | multicenter, prospective, observational | 249 | 73 | Seventy-three (29%) patients failed to wean, with 46 (18%) failing the first spontaneous breathing trial (SBT) and 39 (16%) failing extubation. At the commencement of weaning, 81 patients (33%) had moderate-to-large pleural effusions. Moderate-to-large pleural effusion was associated with more failures of the first SBT [27 (33%) vs. 19 (11%), p < 0.001], more weaning failures [37 (47%) vs. 36 (22%), p < 0.001], less ventilator-free days at day 28 [21 (5-24) vs. 23 (16-26), p = 0.01], and higher mortality at day 28 [14 (17%) vs. 14 (8%), p = 0.04]. The relationship between pleural effusion and weaning failure maintained in multivariable and sensitivity analyses. In patients with difficult weaning, there was no correlation between short-term (48-hour) fluid balance changes and the growth of interpleural distance. |
| Dres M ^64^ | 2018 | France | single-center, prospective, observational | 76 | 28 | Seventy-six individuals were assessed, with 48 (63%) passing and 28 (37%) failing the spontaneous breathing test. The best twitch pressure and thickening fraction criteria for predicting failure in the spontaneous breathing experiment were 7.2 cmH2O and 25.8%, respectively. The receiver operating characteristics curves for twitch pressure and thickening fraction were 0.80 (95% CI 0.70-0.89) and 0.82 (95% CI 0.73-0.93), respectively. The receiver operating characteristic curves were comparable (p = 0.83). A twitch pressure value of less than 11 cmH2O (the conventional cutoff for diaphragm dysfunction) predicted failure of the spontaneous breathing trial with a sensitivity of 89% (95% CI 72-98%) and specificity of 45% (95% CI 30-60%). |
| Loizou CP ^65^ | 2018 | Greece | single-center, prospective, observational | 27 | __ | Cycle duration, excursion, inspiration time, slope, relaxation time, relaxation rate, and MRR were measured using mean ± standard deviation and median (interquartile range (IQR)) values. In addition, the percentage standard error of the mean (%SEM) and percentage of mean absolute error (%MAE) were calculated. Computed the semi-automated ultrasound MRR measurements on all NRES/RES images, using the proposed system (MRR_SAUS = 3.94 ± 0.91/4.98 ± 1.98 [1/sec],%SEM = 5.3/6.31), and compared them with the manual measurements made by a clinical expert (MRR_MUS = 2.36 ± 1.19/5.8 ± 2.1 [1/sec],%SEM = 11.6/11.9 [1/sec]) and those made by a reference manual method (MRR_MB = 3.93 ± 0.89/3.73 ± 0.52 [1/sec],%SEM = 5.2/5.65). We use the Wilcoxon rank sum test to compare the manual (MB and MUS) and SAUS measures of the MRR in all instances studied. MRR_SAUS and MRR_MB readings were not substantially different across NRES and RES participants (p = 0.62/p = 0.58) with a high correlation (ρ = 0.96/ρ =0.83), but were significantly different from the clinical expert's MRR_MUS measurements. It should also be emphasized that the reference technique for measuring MRR is the MRR_MB, which was found in this investigation to be non-statistically substantially different from the suggested semi-automated approach. |
| Khan MT ^66^ | 2018 | Pakistan | single-center, prospective, observational | 90 | 28 | Our study comprised 90 patients: 54 (60%) were men and 36 (40%) were women. The participants' average age was 55 ± 16 years (ranging from 19 to 83 years). 62 patients (68.9%) were effectively weaned. The average DE was 1.44 ± 0.26 cm, and the average RSBI was 56.88 ± 8.30 in all cases. Successful weaning patients had a mean DE of 1.51 ± 0.26 cm and RSBI of 54.05 ± 7.00. The stronger the DE value, the higher the weaning success rate; the lower the RSBI value, the higher the weaning success rate. DE and RSBI had AUCs of 0.795 and 0.815, respectively (p < 0.0001). |
| Tenza-Lozano E ^67^ | 2018 | Spain | single-center, prospective, observational | 69 | 25 | Modified LUS (LUSm) demonstrated good-moderate discriminative power for successful weaning and extubation. TI was more sensitive but less specific in predicting effective weaning (AUC 0.71; ideal sensitivity and specificity 0.93 and 0.48) and successful extubation (AUC 0.76; optimal sensitivity and specificity 0.93 and 0.58). The area under the ROC curve for predicting weaning success was 0.83 when both ultrasound parameters were combined. |
| Theerawit P ^68^ | 2018 | Thailand | single-center, prospective, observational | 62 | 11 | A total of 62 patients were evaluated. The weaning success group had substantially higher mean TPIAdia (1.27 ± 0.38 s; left, 1.14 ± 0.37 s) than the weaning failure group (0.97 ± 0.43 s; left, 0.85 ± 0.39 s) (P < 0.05). A TPIAdia of >0.8s predicts weaning success with 92, 46, 89, and 56% sensitivity, specificity, positive predictive value, and negative predictive value, respectively. Within 48 hours, patients with diaphragmatic inspiratory excursion, DTD, and TFdi required reintubation. The P values were 0.047, 0.021, and 0.028, with areas under the receiver operating characteristic curve of 0.716, 0.805, and 0.784, respectively. |
| Pirompanich P ^69^ | 2018 | Thailand | single-center, prospective, observational | 34 | 9 | The 34 patients enrolled had an average age of 66.5 (±13.5) years. There were 25 patients who were successful at weaning and 9 people who failed. The receiver operating characteristic curves for the right and left DTF, as well as the RSBI, for predicting effective weaning were 0.951, 0.700, and 0.709, respectively. The most reliable cutoff value for predicting effective weaning was right DTF ≥26% (96% sensitivity, 68% specificity, 89% positive predictive value, and 86% negative predictive value). Combining right DTF ≥ 26% and RSBI ≤ 105 boosted specificity to 78% while significantly decreasing sensitivity to 92%. Intra-observer correlation rose dramatically to about 0.9 in the first ten cases, then modestly increased after that. |
| Huang D ^70^ | 2017 | China | single-center, prospective, observational | 40 | 30 | Forty patients were enrolled and evaluated, divided into two groups: those with US-diagnosed diaphragm dysfunction (30/40; 75%) and those without (10/40; 25%). Patients with DD had substantially higher mechanical ventilation duration (536.4±377.05 vs. 250±109.02 hours, P=0.02) and weaning time (425.9±268.31 vs. 216.0±134.22 hours, P=0.002) compared to those without DD. Patients with DD had a greater rate of weaning failure than those without it (24/30 vs. 4/10, P=0.017). The receiver operating characteristic (ROC) curve analysis revealed that the optimal cut-off values to predict weaning success were >10.7 mm for the right diaphragmatic movement and >21.32 mm/s for the right diaphragmatic velocity of contraction; the AUROC were 0.839 (95% CI, 0.689-0.936) and 0.833 (95% CI, 0.682-0.932), respectively. The sensitivity, specificity, positive and negative likelihood ratios for predicting weaning success were 83.33% vs. 66.67%, 75.00% vs. 92.86%, 3.33 vs. 9.33, and 0.22 vs. 0.36 for diaphragmatic contraction velocity and right diaphragmatic movement, respectively. |
| Luo L ^71^ | 2017 | China | single-center, prospective, observational | 60 | 14 | Among 60 patients, 29 experienced respiratory failure within 48 hours, and 14 were re-intubated or died within a week. A multivariate logistic regression analysis found that respiratory failure was linked with E/Ea (average) following SBT [odds ratio (OR) 1.450, 95% confidence intervals (CI) 1.092-1.926, P = 0.01] and left ventricular ejection fraction. After SBT, the AUC of E/Ea was 0.789. A cut-off value of ≥12.5 resulted in the maximum diagnostic accuracy, with sensitivity and specificity of 72.4% and 77.4%, respectively. In the respiratory failure subgroup, only DE following SBT was linked to re-intubation (OR 0.690, CI 0.499-0.953, P = 0.024). The AUC of DE following SBT was 0.805, and a cut-off value ≤ 12.6mm demonstrated the greatest diagnostic accuracy with 80% sensitivity and specificity (68.4%). |
| Farghaly S ^72^ | 2017 | Egipty | single-center, prospective, observational | 54 | 14 | Out of 54 patients, 14 (25.9%) failed extubation. Successful extubation was associated with considerably larger diaphragmatic excursion, Tdi at end inspiration and end expiration, and DTF% compared to unsuccessful extubation (p<0.05). Diaphragmatic measures associated with successful extubation had cutoff values of ≥10.5mm for diaphragmatic excursion, ≥21mm for Tdi at end inspiration, ≥10.5mm for Tdi at end expiration, and ≥34.2% for DTF%, resulting in 87.5%, 77.5%, 80%, and 90% sensitivity and 71.5%, 86.6%, 50%, and 64.3% specificity. Combining diaphragmatic excursion ≥10.5mm and Tdi at end inspiration ≥21mm reduced sensitivity to 64.9% while increasing specificity to 100%. Rapid shallow breathing index (RSBI) <105 showed 90% sensitivity but only 18.7% specificity. |
| Samanta S ^72^ | 2017 | India | single-center, prospective, observational | 64 | 20 | The study comprised 64 (male: female, 40:24) medical (55/64, 86%) patients. Sepsis of lung origin (65.5%) was the leading cause of MV. The SW and CW groups had 33 and 31 patients, respectively. DTf predicts SW with a cutoff of ≥25.5, 26.5, 25.5, and 24.5 for 2, 4, and 6 NPTs and T-piece, with a ROC AUC of ≥0.90. At NPT of 2, DTf exhibited the greatest sensitivity of 97% and specificity of 81% (ROC AUC (CI), 0.91 (0.84-0.99); p < 0.001]. |
| Hayat A ^73^ | 2017 | Pakistan | single-center, prospective, observational | 100 | 24 | Out of 100 instances, 76 patients weaned successfully, whereas 24 weaned unsuccessfully. Out of 67 instances with a diaphragmatic excursion of 1.2 cm or more, 60 (89.55%) were successfully weaned, whereas 7 (10.45%) were unsuccessful. Weaning was effective in 17 of 33 instances (51.5%) with an excursion of less than 1.2 cm, whereas failure occurred in 16 (48.48%). At this threshold (1.2 cm), the sensitivity and specificity for effective weaning were 78.95% and 70.83%, respectively. The positive and negative likelihood ratios (LRs) for these values are 2.70 and 0.29, respectively. The positive predictive value was 82.35 percent, while the negative predictive value was 60.0%. |
| Ruiz-Bailén M ^74^ | 2017 | Spain | single-center, prospective, observational | 181 | 161 | In group A (patients who could not endure a T-tube trial), they found an increase in the E/E' ratio (6.32±0.77 vs 15.2±6.65; P=.0001) and a deterioration of strain (S) and strain rate (SR) (-13.6±1.80 vs -11.88±5.6, P=.0001; and -1.3±1.28 vs -0.95±0.38, P=.0001). The E/E' ratio did not alter during stress echocardiography for patients who successfully weaned from mechanical breathing (7.41±0.43 vs 8.38±4.57, P=.001). This group showed higher peak velocity of the S wave and SR (-16.11±08.72 vs -19.89±5.62 and -1.48±0.23 vs -1.59±0.21, P=.001). In 42 patients with weaning failure, dobutamine echocardiography revealed a higher E/E' ratio (7.41±0.43 vs 15.98±7.98; P=.0001) and worsening of S (-15.41±09.56 vs -12.72±6.55; P=.0001) and SR (-1.41±0.78 vs -1.22±0.65; P=.0001). |
| Grosu HB ^75^ | 2017 | USA | multicenter, prospective, observational | 57 | 16 | Of the 57 participants who had both diaphragm measures at 72 hours, 16 died, 33 were extubated, and 8 required tracheostomy. Only 14 participants received mechanical ventilation for one week, while two had it for two and three weeks. Females exhibited a considerably thinner baseline TDI (P =.008). At 72 hours, TDI had dropped in 84% of the individuals. We discovered no significant relationship between rate of thinning and gender (P =.68), COPD diagnosis (P =.36), current smoking (P =.85), or pleural effusion (P =.83). Lower baseline TDI was linked with an increased chance of extubation: 12.5% greater for every 0.01-cm drop in TDI (hazard ratio 0.875, 95% CI 0.80-0.96, P =.003). Lower baseline TDI was linked with an increased chance of extubation: 12.5% greater for every 0.01-cm drop in TDI (hazard ratio 0.875, 95% CI 0.80-0.96, P =.003). Each 0.01-cm drop in TDI at 72 hours increased the risk of extubation by 17% (hazard ratio 0.83, 95% CI 0.70-0.99, P =.041). |
| Lu Z ^76^ | 2016 | China | single-center, prospective, observational | 41 | __ | The study comprised 41 participants (24 men) aged 62.2 ± 15.9 years. The prevalence of ultrasonographic diaphragmatic dysfunction (defined as diaphragmatic thickening fraction < 20% with inspiration) was 34.1% (n = 14). Diaphragmatic dysfunction was associated with increased ventilation duration (293.4 ± 194.8 vs 145.1 ± 101.3 h, P =.02) and ICU stay (29.2 ± 11.4 vs 22.4 ± 7.7 d, P =.03) compared to those without the condition. |
| Carrie C ^77^ | 2016 | France | single-center, prospective, observational | 67 | 37 | The feasibility rate for ultrasound measurements was 63%. Patients who successfully weaned on their first try had substantially higher mean MDE values (4.1 ± 2.1 versus 3 ± 1.8 cm, P = 0.04). Using a threshold of MDE ≤ 2.7 cm, the sensitivity and specificity of diaphragmatic ultrasonography in predicting weaning failure were 59% [39-77%] and 71% [57-82%], with an AUC of 0.65 [0.51-0.78]. There was no statistically significant difference between MDE values and MRC scores in predicting weaning failure (P = 0.73). |
| Flevari A ^78^ | 2016 | Grecee | single-center, prospective, observational | 27 | __ | Thirteen male and fourteen female patients were included. DEx [median and interquartile range, mm] was 14 (8.5-22) for the right hemidiaphragm (RDEx) and 12 (7-23) for the left. They discovered no difference in DEx between the sexes. LDEx at a cut-off of 10 mm was the most accurate predictor of weaning success (sensitivity 86%, specificity 85%, Negative Predictive Value 94%). The ideal cut-off values, calculated by the area under the receiver operating characteristic curve, were 10 mm for RDEx, 7 mm for LDEx, 57 breaths/min/L for RSBI, and -20 cmH2O for Pimax. |
| Konomi I ^79^ | 2016 | Greece | single-center, prospective, observational | 42 | 15 | Fifteen patients failed to wean. Weaning failure was substantially linked with left ventricular diastolic impairment (P<0.001). The severity of diastolic dysfunction was substantially linked with BNP levels on MV and at the conclusion of the SBT (P<0.001, r=0.703 and P<0.001, r=0.709). Patients who successfully weaned had lower BNP levels on MV compared to those who did not (361±523 vs. 643±382 ng/l, P=0.008). Diastolic dysfunction was shown to be independently linked with weaning failure (odds ratio [OR] 11.23, confidence interval [CI] 1.16-109.1, P=0.037), followed by respiratory frequency/tidal volume (OR 1.05, CI 1.00-1.10, P=0.048). |
| Spadaro S ^80^ | 2016 | Italy | single-center, prospective, observational | 51 | 17 | We included 51 patients who needed mechanical breathing for more than 48 hours and were ready to undergo an SBT. The majority of the patients, 34 (66%), were effectively weaned off mechanical ventilation. When looking at the 17 patients who failed the weaning attempt, 11 (64%) had to be reconnected to the ventilator during the SBT, three (18%) had to be re-intubated within 48 hours of extubation, and three (18%) needed non-invasive breathing assistance within 48 hours of extubation. The areas under the ROC curves for RSBI and D-RSBI were 0.72 and 0.89, respectively (P = 0.006). |
| Dubé B-P ^81^ | 2015 | French | single-center, prospective, observational | 40 | 13 | The study comprised 40 patients (average age 55 ± 18), with 27 in the SW group and 13 in the WF group. The average SOFA score was 5 ± 3, and the duration of mechanical ventilation on the day of the SBT was 7 ± 3. WF patients exhibited lower Ptr,stim (6.1 ± 0.7 vs 13.6 ± 1.2 cmH2O, p < 0.001), lower TFD (21.2 ± 6 vs 35.6 ± 13%, p < 0.01), lower MRC score (40.3 vs 55.7, p < 0.01), and greater TFIC (26.7 ± 15 vs 10.4 ± 6%, p < 0.01). The areas under the receiver operating characteristic curves for predicting WF were 0.86, 0.84, 0.88, and 0.88 for Ptr,stim, TFD, MRC score, and TFIC, respectively (all p < 0.05). The optimal Ptr,stim, and TFD thresholds for predicting WF were 8.2 cm H2O and 29%, respectively.  Ptr,stim showed a substantial correlation with TFd, MRC score, and TFic (rho = 0.88, 0.55, and -0.90, respectively). Only 46% of patients with DD were successfully withdrawn off the ventilator, compared to all patients without DD (p < 0.001). |
| Ferrari G ^82^ | 2014 | Italy | single-center, prospective, observational | 46 | 17 | There was a substantial difference in diaphragm thickness at TLC and RV in both patients who successfully completed SBT and those who failed. DTF differed considerably between individuals who failed and those who completed SBT. A DTF cutoff value of more than 36% was related with a successful SBT, with a sensitivity of 0.82, specificity of 0.88, positive predictive value (PPV) of 0.92, and negative predictive value (NPV) of 0.75. In comparison, RSBI <105 exhibited a sensitivity of 0.93, specificity of 0.88, PPV of 0.93, and NPV of 0.88 in predicting SBT success. |
| Kim WY ^83^ | 2011 | Korea | single-center, prospective, observational | 82 | 44 | The prevalence of ultrasonographic diaphragmatic dysfunction among the 82 eligible patients was 29% (n = 24). Patients with diaphragmatic dysfunction required more time for weaning (401 [range, 226-612] hours vs. 90 [range, 24-309] hrs, p <.01) and total ventilation (576 [range, 374-850] hrs vs. 203 [range, 109-408] hrs, p <.01) compared to those without. Patients with diaphragmatic dysfunction experienced more primary (20 of 24 vs. 34 of 58, p <.01) and secondary (ten of 20 vs. ten of 46, p =.01) weaning failures compared to those without. The area under the receiver operating characteristics curve for ultrasonographic criteria in predicting weaning failure was comparable to that of the quick shallow breathing index. |

Definitions / abbreviations: SBT: spontaneaus breathing trial; LUS: lung ultrasound score; E/e': ratio between early mitral inflow velocity and mitral annular early diastolic velocity ; DTF: diaphragm thickening fraction; AUC: area under the curve; SWE: Shear wave elastography; LASSO: least absolute shrinkage and selection operator ; SVM: support vector machine; LDE: Left diaphragmatic excursion; RDE: Right diaphragmatic excursion ; RET: right expiratory thickening; RIT: right inspiratory thickening; IWI: integrative weaning index; Tdi: tissue Doppler imaging; EVLW: extra vascular lung water; DE: diaphragmatic excursion; ROC: receiver operating characteristic; TF: thickening fraction; NT pro BNP: N-Terminal Pro–B-Type Natriuretic Peptide; RSBI: rapid shallow breathing index; NPV: negative predictive value; PIMTF: parasternal intercostal muscle thickening fraction; P0.1 negative pressure measured at the airway opening 100 ms after the initiation of an inspiratory effort performed against a closed respiratory circuit; Tfdi: the diaphragm thickening fraction; GI: global inhomogeneity; CoV: front back center of ventilation; RVD: regional ventilation delay; COPD: chronic obstructive pulmonary disease; SOFA: Sequential Organ Failure Assessment; NIV: non invasive ventilation; RIS-i: respiratory insufficiency scale-intubated scores; VISAGE: Glasgow Coma Scale for extubation; C-PEFR: patient's cough peak expiratory flow rate ; RR/DTF: ratio between respiratory rate in relation to the diaphragm thickening fraction; RR/DE: ratio between respiratory rate in relation to the diaphragmatic excursion; VTI: velocity time integral; DLS: diaphragm longitudinal strain; Dtei: diaphragm thickness at end-inspiration; Dtee: diaphragm thickness at end-expiration; PLR: passive leg raise; RA: rectus abdominis ; IO: internal oblique; EO: external oblique ; TA: transversus abdominis; ICUAW: intensive care unit acquired weakness; DW: diaphragmatic weakness; VFDs: ventilator-free days; WIND: Weaning according to a New Definition; MIP: maximum inspiratory pressure; VAS: visual analog scale; CFS: clinical frailty scale; BMI: body max index; RF: rectus femoris; VI: vastus intermedius; TRF: thickness rectus femoris; TVI: thickness vastus intermedius; IC-RDOS: Intensive Care Respiratory Distress Observational Scale; MRC: medical research counsil; ΔSMdi: changes in diaphragm shear modulus; ΔPdi: changes in diaphragm pressure; WiPO: Weaning-induced pulmonary oedema; E: early peak diastolic wave ; A: late peak diastolic wave; LV: left ventricular; Edi: electrical activity of the diaphragm; DSF: diaphragmatic shortening fraction; ΔDE: changes in diaphragmatic excursion; TTE: transthoracic echocardiography ; E/Ea: transmitral to mitral annular early diastolic velocity ratio; IVCmax: vena cava maximum diameter; AECOPD: acute exacerbation of chronic obstructive pulmonary disease; MRR: maximum relaxation rate; NRES: with no resistance; RES: with resistance; TPIAdia: time to peak inspiratory amplitude of the diaphragm; DTD: diaphragmatic thickness difference; TFdi: diaphragm thickening fraction; DD: diaphragmatic dysfunction; DTf: diaphragmatic thickening fraction; NPTs: negative pressure triggers; SW: simple weaning; MDE: maximal diaphragmatic excursion; Dex: each hemidiaphragm; RDEx: right hemidiaphragm; LDEx left hemidiaphragm; TFic: The intercostal thickening fraction; SW: successful weaning; WF: weaning failure; Ptr,stim: anterior magnetic phrenic stimulation; TLC: total lung capacity; RV: residual volume

**References**

1. Song J, Luo Q, Lai X, et al. Combined cardiac, lung, and diaphragm ultrasound for predicting weaning failure during spontaneous breathing trial. *Ann Intensive Care*. 2024;14(1):60. doi:10.1186/s13613-024-01294-2

2. Wang Y, Yi Y, Zhang F, et al. Lung Ultrasound Score as a Predictor of Failure to Wean COVID-19 Elderly Patients off Mechanical Ventilation: A Prospective Observational Study. *Clin Interv Aging*. 2024;Volume 19:313-322. doi:10.2147/CIA.S438714

3. Li C, Liu Y, Dong R, Zhang T, Song Y, Zhang Q. Deep learning radiomics on shear wave elastography and b-mode ultrasound videos of diaphragm for weaning outcome prediction. *Med Eng Phys*. 2024;123:104090. doi:10.1016/j.medengphy.2023.104090

4. Lin H, Yao M, Qin Z, Fu S, Wang H. Predictive values of ultrasonic diaphragm thickening fraction combined with integrative weaning index in weaning patients with mechanical ventilation: a retrospective study. *J Cardiothorac Surg*. 2024;19(1):66. doi:10.1186/s13019-024-02544-x

5. Fritsch SJ, Siemer AG, Dreher M, Simon TP, Marx G, Bickenbach J. Diaphragm ultrasound in patients with prolonged weaning from mechanical ventilation. *Quant Imaging Med Surg*. 2024;14(5):3248-3263. doi:10.21037/qims-23-1712

6. Mustafi SM, Talwar V, Krishna B, Rajpal M. Assessment of Extravascular Lung Water Using Lung Ultrasound in Critically Ill Patients Admitted to Intensive Care Unit. *Indian Journal of Critical Care Medicine*. 2024;28(2):165-169. doi:10.5005/jp-journals-10071-24635

7. Sabetian G, Mackie M, Asmarian N, et al. Ultrasonographic evaluation of diaphragm thickness and excursion: correlation with weaning success in trauma patients: prospective cohort study. *J Anesth*. 2024;38(3):354-363. doi:10.1007/s00540-024-03321-9

8. Xin S, Li Y, Liu R, Liu X, Cai S. Tissue Doppler imaging of the diaphragm and outcome of weaning from mechanical ventilation. *Australas J Ultrasound Med*. 2024;27(3):159-166. doi:10.1002/ajum.12389

9. Boscolo A, Sella N, Pettenuzzo T, et al. Diaphragm Dysfunction Predicts Weaning Outcome after Bilateral Lung Transplant. *Anesthesiology*. 2024;140(1):126-136. doi:10.1097/ALN.0000000000004729

10. Hyun J, Kim A ram, Lee SE, Kim MS. B-lines by lung ultrasound as a predictor of re-intubation in mechanically ventilated patients with heart failure. *Front Cardiovasc Med*. 2024;11:1351431. doi:10.3389/fcvm.2024.1351431

11. Pu H, Doig GS, Lv Y, et al. Modifiable risk factors for ventilator associated diaphragmatic dysfunction: a multicenter observational study. *BMC Pulm Med*. 2023;23(1):343. doi:10.1186/s12890-023-02633-y

12. He G, Han Y, Zhan Y, Yao Y, Zhou H, Zheng X. The combined use of parasternal intercostal muscle thickening fraction and P0.1 for prediction of weaning outcomes. *Heart & Lung*. 2023;62:122-128. doi:10.1016/j.hrtlng.2023.07.002

13. Huang D, Song F, Luo B, et al. Using automatic speckle tracking imaging to measure diaphragm excursion and predict the outcome of mechanical ventilation weaning. *Crit Care*. 2023;27(1):18. doi:10.1186/s13054-022-04288-3

14. Varón-Vega F, Giraldo-Cadavid LF, Uribe AM, et al. Utilization of spontaneous breathing trial, objective cough test, and diaphragmatic ultrasound results to predict extubation success: COBRE-US trial. *Crit Care*. 2023;27(1):414. doi:10.1186/s13054-023-04708-y

15. Ramaswamy A, Kumar R, Ish P, et al. Prediction of Weaning Outcome from Mechanical Ventilation Using Ultrasound Assessment of Parasternal Intercostal Muscle Thickness. *Indian Journal of Critical Care Medicine*. 2023;27(10):704-708. doi:10.5005/jp-journals-10071-24548

16. Laguado-Nieto MA, Roberto-Avilán SL, Naranjo-Junoy F, et al. Diaphragmatic Dynamics and Thickness Parameters Assessed by Ultrasonography Predict Extubation Success in Critically Ill Patients. *Clin Med Insights Circ Respir Pulm Med*. 2023;17:11795484231165940. doi:10.1177/11795484231165940

17. Joussellin V, Bonny V, Spadaro S, et al. Lung aeration estimated by chest electrical impedance tomography and lung ultrasound during extubation. *Ann Intensive Care*. 2023;13(1):91. doi:10.1186/s13613-023-01180-3

18. Rajbanshi L, Bajracharya A, Devkota D. Prediction of Successful Spontaneous Breathing Trial and Extubation of Trachea by Lung Ultrasound in Mechanically Ventilated Patients in Intensive Care Unit. *Indian Journal of Critical Care Medicine*. 2023;27(7):482-487. doi:10.5005/jp-journals-10071-24487

19. Raj I, Kumar Nagaiah S. Ultrasonography of Diaphragm to Predict Extubation Outcome. *Cureus*. 2023;15(3):e36514. doi:10.7759/cureus.36514

20. Hirolli D, Srinivasaiah B, Muthuchellappan R, Chakrabarti D. Clinical Scoring and Ultrasound-Based Diaphragm Assessment in Predicting Extubation Failure in Neurointensive Care Unit: A Single-Center Observational Study. *Neurocrit Care*. 2023;39(3):690-696. doi:10.1007/s12028-023-01695-4

21. Eksombatchai D, Sukkratok C, Sutherasan Y, Junhasavasdikul D, Theerawit P. The ratio of respiratory rate to diaphragm thickening fraction for predicting extubation success. *BMC Pulm Med*. 2023;23(1):109. doi:10.1186/s12890-023-02392-w

22. Er B, Mızrak B, Aydemir A, et al. Is diaphragm ultrasound better than rapid shallow breathing index for predicting weaning in critically ill elderly patients? *Tuberk Toraks*. 2023;71(3):197-202. doi:10.5578/tt.20239701

23. Al Tayar AS, Abdelshafey EE. Diaphragm Electromyography Versus Ultrasonography in the Prediction of Mechanical Ventilation Liberation Outcome. *Respir Care*. 2022;67(11):1437-1442. doi:10.4187/respcare.09779

24. Allam MGIM. Comparative Study between the use of Ultrasonic Criteria of Weaning versus the Conventional Criteria of Weaning in Post-traumatic Acute Respiratory Distress Syndrome Patients who were Ventilated for a long Time. *The Open Anesthesia Journal*. 2023;17(1). doi:10.2174/18743218-v17-e230913-2023-12

25. Xu Q, Yang X, Qian Y, et al. Comparison of assessment of diaphragm function using speckle tracking between patients with successful and failed weaning: a multicentre, observational, pilot study. *BMC Pulm Med*. 2022;22(1):459. doi:10.1186/s12890-022-02260-z

26. Song J, Qian Z, Zhang H, et al. Diaphragmatic ultrasonography-based rapid shallow breathing index for predicting weaning outcome during a pressure support ventilation spontaneous breathing trial. *BMC Pulm Med*. 2022;22(1):337. doi:10.1186/s12890-022-02133-5

27. Saravanan R, Nivedita K, Karthik K, Venkatraman R. Role of diaphragm ultrasound in weaning mechanically ventilated patients: A prospective observational study. *Indian J Anaesth*. 2022;66(8):591-598. doi:10.4103/ija.ija_229_22

28. Shamil P, Sen M, Kumar R, et al. Prediction of Weaning Outcome from Mechanical Ventilation Using Diaphragmatic Rapid Shallow Breathing Index. *Indian Journal of Critical Care Medicine*. 2022;26(9):1000-1005. doi:10.5005/jp-journals-10071-24316

29. Lalwani LK, Govindagoudar MB, Singh PK, Sharma M, Chaudhry D. The role of diaphragmatic thickness measurement in weaning prediction and its comparison with rapid shallow breathing index: a single-center experience. *Acute and Critical Care*. 2022;37(3):347-354. doi:10.4266/acc.2022.00108

30. Kundu R, Baidya D, Anand R, Maitra S, Soni K, Subramanium R. Integrated ultrasound protocol in predicting weaning success and extubation failure: a prospective observational study. *Anaesthesiol Intensive Ther*. 2022;54(2):156-163. doi:10.5114/ait.2022.115351

31. Amara V, Natarajan S, Maddani SS, Chaudhuri S, Vishwas P. Evaluation of Abdominal Expiratory Muscle Thickness Pattern, Diaphragmatic Excursion, and Lung Ultrasound Score in Critically Ill Patients and Their Association with Weaning Patterns: A Prospective Observational Study. *Indian Journal of Critical Care Medicine*. 2022;26(3):307-313. doi:10.5005/jp-journals-10071-24125

32. Vetrugno L, Orso D, Corradi F, et al. Diaphragm ultrasound evaluation during weaning from mechanical ventilation in COVID-19 patients: a pragmatic, cross-section, multicenter study. *Respir Res*. 2022;23(1):210. doi:10.1186/s12931-022-02138-y

33. Bertoni M, Piva S, Beretta A, et al. Occurrence and Effects on Weaning From Mechanical Ventilation of Intensive Care Unit Acquired and Diaphragm Weakness: A Pilot Study. *Front Med (Lausanne)*. 2022;9:930262. doi:10.3389/fmed.2022.930262

34. Pierrakos C, Lieveld A, Pisani L, et al. A Lower Global Lung Ultrasound Score Is Associated with Higher Likelihood of Successful Extubation in Invasively Ventilated COVID-19 Patients. *Am J Trop Med Hyg*. 2021;105(6):1490-1497. doi:10.4269/ajtmh.21-0545

35. Li S, Chen Z, Yan W. Application of bedside ultrasound in predicting the outcome of weaning from mechanical ventilation in elderly patients. *BMC Pulm Med*. 2021;21(1):217. doi:10.1186/s12890-021-01605-4

36. Helmy MA, Magdy Milad L, Osman SH, Ali MA, Hasanin A. Diaphragmatic excursion: A possible key player for predicting successful weaning in patients with severe COVID-19. *Anaesth Crit Care Pain Med*. 2021;40(3):100875. doi:10.1016/j.accpm.2021.100875

37. Fossat G, Daillet B, Desmalles E, Boulain T. Does diaphragm ultrasound improve the rapid shallow breathing index accuracy for predicting the success of weaning from mechanical ventilation? *Australian Critical Care*. 2022;35(3):233-240. doi:10.1016/j.aucc.2021.05.008

38. Dres M, Similowski T, Goligher EC, et al. Dyspnoea and respiratory muscle ultrasound to predict extubation failure. *European Respiratory Journal*. 2021;58(5):2100002. doi:10.1183/13993003.00002-2021

39. Dres M, Rozenberg E, Morawiec E, et al. Diaphragm dysfunction, lung aeration loss and weaning-induced pulmonary oedema in difficult-to-wean patients. *Ann Intensive Care*. 2021;11(1):99. doi:10.1186/s13613-021-00886-6

40. Cammarota G, Boniolo E, Santangelo E, et al. Diaphragmatic Kinetics Assessment by Tissue Doppler Imaging and Extubation Outcome. *Respir Care*. 2021;66(6):983-993. doi:10.4187/respcare.08702

41. Trifi A, Abdellatif S, Ben Lamine F, Abdennebi C, Touil Y, Ben Lakhal S. Ultrasound assessment of the diaphragm during the first days of mechanical ventilation compared to spontaneous respiration: a comparative study. *Tunis Med*. 99(11):1055-1065.

42. Er B, Simsek M, Yildirim M, et al. Association of baseline diaphragm, rectus femoris and vastus intermedius muscle thickness with weaning from mechanical ventilation. *Respir Med*. 2021;185:106503. doi:10.1016/j.rmed.2021.106503

43. Gok F, Mercan A, Kilicaslan A, Sarkilar G, Yosunkaya A. Diaphragm and Lung Ultrasonography During Weaning From Mechanical Ventilation in Critically Ill Patients. *Cureus*. 2021;13(5):e15057. doi:10.7759/cureus.15057

44. Whebell S, Sane S, Naidu S, White H. Use of Ultrasound to Determine Changes in Diaphragm Mechanics During A Spontaneous Breathing Trial. *J Intensive Care Med*. 2021;36(9):1044-1052. doi:10.1177/0885066620943164

45. Porto DB, Beltrão BA, Medeiros FS, Pellegrini JAS, Boniatti MM. Preload Independence Is Associated with Extubation Failure in Simple Weaning Patients: A Multicenter Cohort Study. *Am J Respir Crit Care Med*. 2021;203(7):916-918. doi:10.1164/rccm.202008-3250LE

46. Elshazly MI, Kamel KM, Elkorashy RI, Ismail MS, Ismail JH, Assal HH. Role of Bedside Ultrasonography in Assessment of Diaphragm Function as a Predictor of Success of Weaning in Mechanically Ventilated Patients. *Tuberc Respir Dis (Seoul)*. 2020;83(4):295-302. doi:10.4046/trd.2020.0045

47. Fossé Q, Poulard T, Niérat MC, et al. Ultrasound shear wave elastography for assessing diaphragm function in mechanically ventilated patients: a breath-by-breath analysis. *Crit Care*. 2020;24(1):669. doi:10.1186/s13054-020-03338-y

48. Goudelin M, Champy P, Amiel JB, et al. Left ventricular overloading identified by critical care echocardiography is key in weaning-induced pulmonary edema. *Intensive Care Med*. 2020;46(7):1371-1381. doi:10.1007/s00134-020-06061-y

49. Bouhemad B, Mojoli F, Nowobilski N, et al. Use of combined cardiac and lung ultrasound to predict weaning failure in elderly, high-risk cardiac patients: a pilot study. *Intensive Care Med*. 2020;46(3):475-484. doi:10.1007/s00134-019-05902-9

50. Krishnakumar M, Muthuchellappan R, Chakrabarti D. Diaphragm Function Assessment During Spontaneous Breathing Trial in Patients with Neuromuscular Diseases. *Neurocrit Care*. 2021;34(2):382-389. doi:10.1007/s12028-020-01141-9

51. Xia J, Qian CY, Yang L, et al. Influence of lung aeration on diaphragmatic contractility during a spontaneous breathing trial: an ultrasound study. *J Intensive Care*. 2019;7(1):54. doi:10.1186/s40560-019-0409-x

52. Zhang X, Yuan J, Zhan Y, et al. Evaluation of diaphragm ultrasound in predicting extubation outcome in mechanically ventilated patients with COPD. *Irish Journal of Medical Science (1971 -)*. 2020;189(2):661-668. doi:10.1007/s11845-019-02117-1

53. Varón-Vega F, Hernández Á, López M, et al. Utilidad de la ecografía diafragmática para predecir el éxito en la extubación. *Med Intensiva*. 2021;45(4):226-233. doi:10.1016/j.medin.2019.10.007

54. Eltrabili HH, Hasanin AM, Soliman MS, Lotfy AM, Hamimy WI, Mukhtar AM. Evaluation of Diaphragmatic Ultrasound Indices as Predictors of Successful Liberation From Mechanical Ventilation in Subjects With Abdominal Sepsis. *Respir Care*. 2019;64(5):564-569. doi:10.4187/respcare.06391

55. Vivier E, Muller M, Putegnat JB, et al. Inability of Diaphragm Ultrasound to Predict Extubation Failure. *Chest*. 2019;155(6):1131-1139. doi:10.1016/j.chest.2019.03.004

56. Ferré A, Guillot M, Lichtenstein D, et al. Lung ultrasound allows the diagnosis of weaning-induced pulmonary oedema. *Intensive Care Med*. 2019;45(5):601-608. doi:10.1007/s00134-019-05573-6

57. González-Aguirre JE, Rivera-Uribe CP, Rendón-Ramírez EJ, Cañamar-Lomas R, Serna-Rodríguez JA, Mercado-Longoría R. Pulmonary Ultrasound and Diaphragmatic Shortening Fraction Combined Analysis for Extubation-Failure-Prediction in Critical Care Patients. *Arch Bronconeumol*. 2019;55(4):195-200. doi:10.1016/j.arbres.2018.09.015

58. Tongyoo S, Thomrongpairoj P, Permpikul C. Efficacy of echocardiography during spontaneous breathing trial with low‐level pressure support for predicting weaning failure among medical critically ill patients. *Echocardiography*. 2019;36(4):659-665. doi:10.1111/echo.14306

59. Rittayamai N, Hemvimon S, Chierakul N. The evolution of diaphragm activity and function determined by ultrasound during spontaneous breathing trials. *J Crit Care*. 2019;51:133-138. doi:10.1016/j.jcrc.2019.02.016

60. Haji K, Haji D, Canty DJ, Royse AG, Green C, Royse CF. The impact of heart, lung and diaphragmatic ultrasound on prediction of failed extubation from mechanical ventilation in critically ill patients: a prospective observational pilot study. *Crit Ultrasound J*. 2018;10(1):13. doi:10.1186/s13089-018-0096-1

61. Abbas A, Embarak S, Walaa M, Lutfy S. Role of diaphragmatic rapid shallow breathing index in predicting weaning outcome in patients with acute exacerbation of COPD. *Int J Chron Obstruct Pulmon Dis*. 2018;Volume 13:1655-1661. doi:10.2147/COPD.S161691

62. Palkar A, Narasimhan M, Greenberg H, et al. Diaphragm Excursion-Time Index. *Chest*. 2018;153(5):1213-1220. doi:10.1016/j.chest.2018.01.007

63. Razazi K, Boissier F, Neuville M, et al. Pleural effusion during weaning from mechanical ventilation: a prospective observational multicenter study. *Ann Intensive Care*. 2018;8(1):103. doi:10.1186/s13613-018-0446-y

64. Dres M, Goligher EC, Dubé BP, et al. Diaphragm function and weaning from mechanical ventilation: an ultrasound and phrenic nerve stimulation clinical study. *Ann Intensive Care*. 2018;8(1):53. doi:10.1186/s13613-018-0401-y

65. Loizou CP, Matamis D, Minas G, et al. A New Method for Diaphragmatic Maximum Relaxation Rate Ultrasonographic Measurement in the Assessment of Patients With Diaphragmatic Dysfunction. *IEEE J Transl Eng Health Med*. 2018;6:1-10. doi:10.1109/JTEHM.2018.2868671

66. Khan MT, Munawar K, Hussain SW, et al. Comparing Ultrasound-based Diaphragmatic Excursion with Rapid Shallow Breathing Index as a Weaning Predictor. *Cureus*. 2018;10(12):e3710. doi:10.7759/cureus.3710

67. Tenza-Lozano E, Llamas-Alvarez A, Jaimez-Navarro E, Fernández-Sánchez J. Lung and diaphragm ultrasound as predictors of success in weaning from mechanical ventilation. *Crit Ultrasound J*. 2018;10(1):12. doi:10.1186/s13089-018-0094-3

68. Theerawit P, Eksombatchai D, Sutherasan Y, Suwatanapongched T, Kiatboonsri C, Kiatboonsri S. Diaphragmatic parameters by ultrasonography for predicting weaning outcomes. *BMC Pulm Med*. 2018;18(1):175. doi:10.1186/s12890-018-0739-9

69. Pirompanich P, Romsaiyut S. Use of diaphragm thickening fraction combined with rapid shallow breathing index for predicting success of weaning from mechanical ventilator in medical patients. *J Intensive Care*. 2018;6(1):6. doi:10.1186/s40560-018-0277-9

70. Huang D, Ma H, Zhong W, et al. Using M-mode ultrasonography to assess diaphragm dysfunction and predict the success of mechanical ventilation weaning in elderly patients. *J Thorac Dis*. 2017;9(9):3177-3186. doi:10.21037/jtd.2017.08.16

71. Luo L, Li Y, Chen X, et al. Different effects of cardiac and diaphragm function assessed by ultrasound on extubation outcomes in difficult-to-wean patients: a cohort study. *BMC Pulm Med*. 2017;17(1):161. doi:10.1186/s12890-017-0501-8

72. Farghaly S, Hasan AA. Diaphragm ultrasound as a new method to predict extubation outcome in mechanically ventilated patients. *Australian Critical Care*. 2017;30(1):37-43. doi:10.1016/j.aucc.2016.03.004

73. Hayat A, Khan A, Khalil A, Asghar A. Diaphragmatic Excursion: Does it Predict Successful Weaning from Mechanical Ventilation? *J Coll Physicians Surg Pak*. 2017;27(12):743-746.

74. Ruiz-Bailén M, Cobo-Molinos J, Castillo-Rivera A, et al. Stress echocardiography in patients who experienced mechanical ventilation weaning failure. *J Crit Care*. 2017;39:66-71. doi:10.1016/j.jcrc.2017.01.004

75. Grosu HB, Ost DE, Lee YI, et al. Diaphragm Muscle Thinning in Subjects Receiving Mechanical Ventilation and Its Effect on Extubation. *Respir Care*. 2017;62(7):904-911. doi:10.4187/respcare.05370

76. Lu Z, Xu Q, Yuan Y, Zhang G, Guo F, Ge H. Diaphragmatic Dysfunction Is Characterized by Increased Duration of Mechanical Ventilation in Subjects With Prolonged Weaning. *Respir Care*. 2016;61(10):1316-1322. doi:10.4187/respcare.04746

77. Carrie C, Gisbert-Mora C, Bonnardel E, et al. Ultrasonographic diaphragmatic excursion is inaccurate and not better than the MRC score for predicting weaning-failure in mechanically ventilated patients. *Anaesth Crit Care Pain Med*. 2017;36(1):9-14. doi:10.1016/j.accpm.2016.05.009

78. Flevari A, Lignos M, Konstantonis D, Armaganidis A. Diaphragmatic ultrasonography as an adjunct predictor tool of weaning success in patients with difficult and prolonged weaning. *Minerva Anestesiol*. 2016;82(11):1149-1157.

79. Konomi I, Tasoulis A, Kaltsi I, et al. Left Ventricular Diastolic Dysfunction—An Independent Risk Factor for Weaning Failure from Mechanical Ventilation. *Anaesth Intensive Care*. 2016;44(4):466-473. doi:10.1177/0310057X1604400408

80. Spadaro S, Grasso S, Mauri T, et al. Can diaphragmatic ultrasonography performed during the T-tube trial predict weaning failure? The role of diaphragmatic rapid shallow breathing index. *Crit Care*. 2016;20(1):305. doi:10.1186/s13054-016-1479-y

81. Dubé BP, Demoule A, Mayaux J, et al. Ultrasonographically diagnosed diaphragmatic dysfunction and weaning failure from mechanical ventilation in critically ill patients. *Intensive Care Med Exp*. 2015;3(S1):A454. doi:10.1186/2197-425X-3-S1-A454

82. Ferrari G, De Filippi G, Elia F, Panero F, Volpicelli G, Aprà F. Diaphragm ultrasound as a new index of discontinuation from mechanical ventilation. *Crit Ultrasound J*. 2014;6(1):8. doi:10.1186/2036-7902-6-8

83. Kim WY, Suh HJ, Hong SB, Koh Y, Lim CM. Diaphragm dysfunction assessed by ultrasonography: Influence on weaning from mechanical ventilation*. *Crit Care Med*. 2011;39(12):2627-2630. doi:10.1097/CCM.0b013e3182266408
